# Off-Target-Based Tumor Fraction Estimation from Targeted Sequencing Shows Concordance with Orthogonal Methods Across Advanced Solid Tumors

**DOI:** 10.3390/ijms27136078

**Published:** 2026-07-07

**Authors:** Samantha O. Hasenleithner, Shilpa Rao, Jian Q. Yu, Yinfei Tan, Fathima Sheriff, Jennifer S. Winn, Hossein Borghaei, Martin J. Edelman, Anshu Giri, Igor Astsaturov, Mariusz Wasik, Philipp J. Jost, Sandra V. Fernandez

**Affiliations:** 1 Division of Oncology, Department of Internal Medicine, Medical University of Graz, 8036 Graz, Austria; samantha.hasenleithner@medunigraz.at (S.O.H.); philipp.jost@medunigraz.at (P.J.J.); 2 Vessel FlexCo, 8042 Graz, Austria; 3 Departments of Pathology, Fox Chase Cancer Center, Philadelphia, PA 19111, USA; shilpa.rao@fccc.edu (S.R.); yinfei.tan@fccc.edu (Y.T.); mariusz.wasik@fccc.edu (M.W.); 4 Department of Diagnostic Imaging, Fox Chase Cancer Center, Philadelphia, PA 19111, USA; michael.yu@fccc.edu; 5 Office of Clinical Research, Fox Chase Cancer Center, Philadelphia, PA 19111, USA; fathima.sheriff@fccc.edu; 6 Department of Hematology/Oncology, Fox Chase Cancer Center, Philadelphia, PA 19111, USA; jennifers.winn@fccc.edu (J.S.W.); hossein.borghaei@fccc.edu (H.B.); martin.edelman@fccc.edu (M.J.E.); anshu.giri@fccc.edu (A.G.); igor.astsaturov@fccc.edu (I.A.)

**Keywords:** ctDNA, cfDNA, ctFraction, circulating tumor fraction, molecular response, mean VAF, mVAF, fragmentomic, Fragle, ichorCNA

## Abstract

Circulating tumor DNA fraction (ctFraction) has emerged as an important biomarker for assessing tumor burden and monitoring treatment response in patients with cancer. In this study, we compared ctFraction estimates generated by ichorCNA, Fragle low-pass whole-genome sequencing (Fragle LP-WGS), Fragle off-target, and OTTER, a proprietary algorithm from Tempus AI. Plasma samples from 33 patients with advanced solid tumors were analyzed using a ctDNA assay targeting 150 cancer-associated genes, and ctFraction estimates generated by the different methods were compared. Fragle off-target demonstrated the highest concordance with Fragle LP-WGS (rho = 0.903), followed by OTTER (rho = 0.698) and ichorCNA (rho = 0.696), while OTTER and ichorCNA showed strong agreement (rho = 0.826). Mean VAF (mVAF) significantly correlated with all ctFraction estimates, with the strongest association observed for ichorCNA (rho = 0.910), followed by OTTER (rho = 0.865), Fragle LP-WGS (rho = 0.680), and Fragle off-target (rho = 0.658). Longitudinal analysis of 20 patients at baseline and after two cycles of treatment demonstrated strong correlations between changes in ctFraction (ΔctFraction) and mean ΔVAF for both ichorCNA and Fragle off-target (r = 0.955 and r = 0.906, respectively). Overall, these findings demonstrate that ctFraction estimates derived from copy-number- and fragmentomic-based approaches show strong concordance across advanced solid tumors and significantly correlate with mVAF, a commonly used measure of ctDNA abundance. Fragle off-target, in particular, provides an efficient strategy for ctFraction estimation directly from existing targeted sequencing data, eliminating the need for additional sequencing. Larger prospective studies are warranted to further evaluate Fragle off-target clinical utility for treatment monitoring and outcome prediction.

## 1. Introduction

Circulating tumor DNA (ctDNA) is a component of circulating cell-free DNA (cfDNA) that is shed by malignant tumors into the blood, urine, pleural fluid, ascites, and saliva. ctDNA, which carries tumor-specific genetic alterations, typically constitutes a small proportion of an individual’s total cfDNA. In healthy individuals, the majority of cfDNA originates from hematopoietic cells, erythrocytes, leukocytes, and endothelial cells, whereas in patients with advanced cancers, ctDNA is derived from both primary tumors and metastatic sites [1]. Compared with cfDNA derived from non-cancer cells, ctDNA is shorter, with a modal fragment size of ~146 bp versus ~166 bp for cfDNA from normal cells [2,3,4,5]. ctDNA has a short half-life, ranging from 16 min to 2.5 h [6,7], enabling real-time assessment of therapeutic response and clinical outcomes, commonly referred to as molecular response [8].

In recent years, ctDNA has gained recognition as a robust biomarker for cancer diagnostics, surveillance, and early detection of relapses, as well as treatment monitoring, showing significant lead times over traditional imaging [9,10,11,12,13,14,15]. Studies monitoring patients with advanced cancers through therapy have shown that ctDNA dynamics correlate with treatment response and may identify responders earlier than clinical or radiological detection [16,17,18,19]. Across multiple different tumor types and treatment modalities, responders typically demonstrate a rapid decline in ctDNA levels within weeks of therapy initiation.

Circulating tumor fraction (ctFraction) represents the proportion of tumor-derived DNA relative to total cfDNA and varies depending on tumor type, location, stage, tumor burden, and response to therapy [16,20,21,22,23]. Several algorithms have been developed to estimate ctFraction from next-generation sequencing (NGS) data, including ichorCNA [24], Fragle [25], and Off-Target Tumor Estimation Routine (OTTER) [26]. ichorCNA estimates ctFraction by detecting large-scale copy number alterations (CNAs) and aneuploidies, but requires low-pass whole-genome sequencing (LP-WGS), making it unsuitable for targeted panels routinely employed in clinical settings, which use deep sequencing rather than LP-WGS [24]. Fragmentomic is an emerging field in liquid biopsies [14,27,28,29,30,31], with Fragle being an ultra-fast, deep-learning-based method recently developed to quantify ctDNA levels directly from cfDNA fragmentomic profiles [25]. Fragle estimates ctDNA levels by analyzing cfDNA fragment length distributions from NGS data, based on the tendency of tumor-derived DNA fragments to be shorter than non-tumor cfDNA fragments. The method can be applied to both low-pass whole-genome sequencing (LP-WGS) data (Fragle LP-WGS) and off-target reads generated from deep targeted sequencing commonly used for tumor profiling (Fragle off-target). OTTER developed by Tempus AI, Inc. (Chicago, IL, USA), utilizes targeted NGS data from assays such as Tempus xF and xF+, integrating somatic variant allele frequencies (VAFs) and CNAs to estimate ctFraction; however, the complete algorithmic methodology is proprietary and not publicly disclosed [26]. VAFs of tumor-related variants (somatic variants) can also be used to approximate ctDNA levels; however, not all tumors harbor mutations covered by a given targeted sequencing panel, and the accuracy of this approach depends on the ability to distinguish germline variants and clonal hematopoiesis of indeterminate potential (CHIP) from true somatic alterations [32,33].

**Figure 1:**
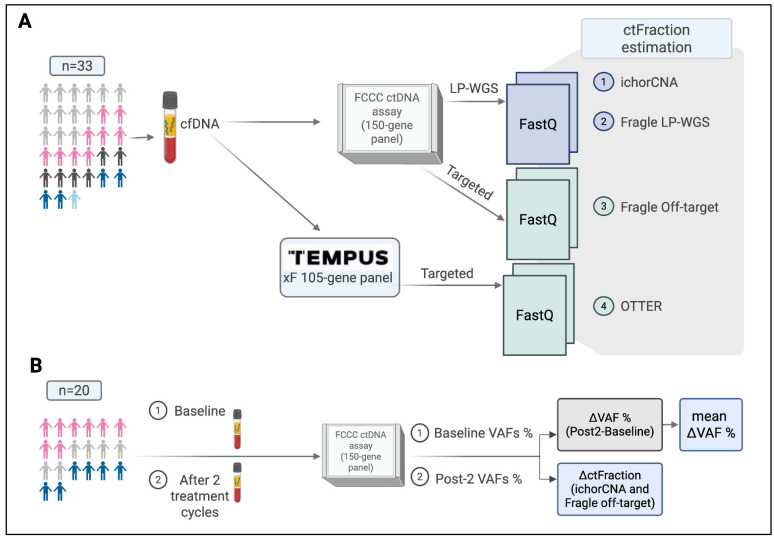
Study overview and workflow. (**A**) Thirty-three patients with diverse solid tumors underwent liquid biopsy analysis. The cfDNA underwent library preparation for the FCCC ctDNA assay and the resulting whole-genome library was subjected to low-pass whole-genome sequencing (LP-WGS), while the enriched library underwent deep targeted sequencing. Raw NGS datasets were analyzed using multiple algorithms to estimate ctFraction. LP-WGS data (purple FastQ files) were analyzed using both ichorCNA and Fragle LP-WGS, whereas FCCC targeted sequencing data (green FastQ files) were analyzed using Fragle off-target. In parallel, cfDNA samples from each patient were sent to Tempus for external ctDNA genomic profiling using the Tempus xF 105-gene assay, and ctFraction estimation by OTTER. Correlation analyses were performed between the different ctFraction estimates and mean variant allele frequency (mVAF) values obtained from tumor profiling by the FCCC assay. (**B**) Twenty patients with diverse solid tumors who had paired baseline and post-treatment liquid biopsy samples available underwent genomic profiling using the FCCC ctDNA assay. Variant allele frequencies (VAFs, %) at baseline and after treatment (following two cycles of therapy, Post-2) were analyzed for each detected variant to determine the change in VAF after treatment (ΔVAF, %). Mean ΔVAF (%) and ΔctFraction were subsequently calculated for each patient to assess treatment response. In both panels, (**A**) and (**B**), when the FCCC ctDNA assay was performed for tumor profiling, both cfDNA and matched gDNA isolated from whole blood underwent library preparation to enable subsequent CHIP and germline filtering. Only somatic variants were used to calculate mean VAF (mVAF) and mean ΔVAF (same as Δ mean VAF). Colored patient icons indicate tumor type: pink, breast cancer; light gray, lung cancer; dark gray (panel (**A**)), patients with melanoma; dark blue, colorectal cancer; light blue (panel (**A**)), patient with esophageal cancer. Created in BioRender, Hasenleithner, S. (2026). https://BioRender.com/xlmdsaf, accessed on 29 June 2026.

In the present study, ctFraction was estimated using ichorCNA, Fragle LP-WGS, and Fragle off-target in a cohort of 33 patients with advanced solid tumors, and these estimates were compared with OTTER (Figure 1A). The FCCC assay, a 150-gene ctDNA-based NGS assay, was used for tumor profiling and ctFraction estimation using the Fragle off-target method, which analyzes off-target reads generated during deep sequencing (Figure 1A). In parallel, separate libraries were prepared for low-pass whole-genome sequencing (LP-WGS), as ichorCNA and Fragle LP-WGS require genome-wide sequencing data for ctFraction estimation and cannot be applied to the deep targeted sequencing data generated by the FCCC ctDNA assay for tumor profiling (Figure 1A). 

Additionally, tumor variants from 20 cancer patients were evaluated at baseline and after two therapy cycles (Post-2) using the FCCC ctDNA assay. Changes in variant allele frequency (VAF) and circulating tumor fraction (ctFraction), as estimated via ichorCNA and Fragle off-target sequencing, were used to assess molecular and treatment-associated responses (Figure 1B).

## 2. Results

### 2.1. Cohort of 33 Patients with Solid Tumors to Study ctFraction

Blood samples from 33 patients with advanced solid tumors (one sample per patient) were used to evaluate ctFraction estimates. Of the 33 patients included in this cohort, 13 had non-small cell lung cancer (10 adenocarcinoma and 3 squamous cell carcinoma), 6 had melanoma, 1 had esophageal cancer, 3 had colon adenocarcinoma, 1 had rectal adenocarcinoma, and 9 had breast cancer (Supplementary Table S1, Pt.1–33). At the time of blood draw for ctDNA analysis, 28 patients had pathological stage IV and 5 had stage III disease; three patients had a history of more than one type of cancer (Supplementary Table S1, Pt.1–33). cfDNA and paired genomic DNA (gDNA, matched normal DNA) extracted from blood were profiled using a validated 150-gene NGS assay, the FCCC ctDNA assay [34]. Genomic profiling was performed using hybridization-captured, adaptor ligation-based libraries to a median unique coverage depth of 4246× reporting SNV and indels. Because both plasma and matched normal samples were analyzed for all patients, germline and CHIP-associated variants could be distinguished from somatic variants (tumor-derived). A total of 18 germline variants, including 14 variants of uncertain significance (VUS), 17 CHIPs, and 156 somatic variants, were identified (Supplementary Tables S1 and S2).

Pathogenic or likely pathogenic germline variants were found in 4 of 33 patients (12.1%), all of which had a VAF around 50%. Two patients—one with lung squamous cell carcinoma (Pt.5) and another (Pt.30) with breast cancer and a history of lung cancer—harbored pathogenic germline *MUTYH* variants. Pt.29, diagnosed with breast cancer, had a pathogenic germline *BRCA1* variant, and Pt.11, with lung adenocarcinoma, carried a pathogenic germline *ATM* variant (Supplementary Table S1).

Ten of the 33 patients (30.3%) harbored between one and three CHIP variants, with VAFs > 0.2% (Supplementary Table S1). Two elderly patients, Pt.18 (86 years old) and Pt.10 (80 years old), exhibited CHIP-associated oncogenic variants at high VAFs (~40%). Pt.18 harbored TP53 and DNMT3A CHIP-associated variants, whereas Pt.10 harbored a DNMT3A CHIP variant (Supplementary Table S1).

**Figure 2:**
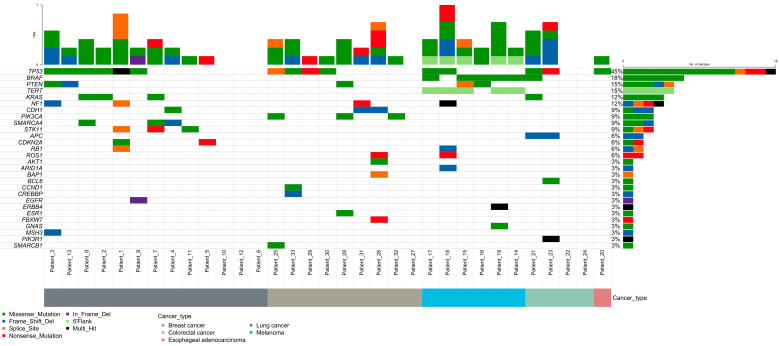
Somatic oncogenic and likely oncogenic variants detected using FCCC ctDNA assay in a cohort of 33 patients with advanced cancers. Top 30 genes affected by oncogenic and likely oncogenic variants as detected by FCCC ctDNA assay.

Somatic variants were detected with the FCCC ctDNA assay in 29 of 33 patients (87.9%); no variants were detected in Pt.10, Pt.12, Pt.22, and Pt.24 (Supplementary Table S2). Out of 156 total detected variants, 77 were classified as oncogenic, likely oncogenic, Tier 1, or Tier 2 according to Cancer KB (Golden Helix) (Supplementary Table S2). As expected, *TP53* was the most frequent mutated gene, with 15 patients (45%) harboring oncogenic mutations (Figure 2). Six patients (18%) harbored *BRAF* variants, which were classified as Tier 1 in four melanoma patients (Pt.14, Pt.15, Pt.16, and Pt.17). Additionally, oncogenic or likely oncogenic *PTEN* variants were detected in five patients (15%; two lung squamous cell carcinoma, two melanoma, and one breast cancer), *TERT* variants in five melanoma patients (15%), and Tier 1 *KRAS* variants in four patients (12%; three lung adenocarcinoma and one colon adenocarcinoma). *KRAS* Gly12Ala, Gly12Asp, and Gly12Val are classified as Tier 1 variants, as these alterations are associated with poorer survival [35]. In lung adenocarcinoma patients, Pt.9 harbored an *EGFR* variant located in the exon 19 tyrosine kinase domain conferring sensitivity to *EGFR* tyrosine kinase inhibitors (TKIs) (Supplementary Table S2). Finally, oncogenic *PIK3CA* variants were identified in three breast cancer patients (Figure 2).

### 2.2. ctFraction Estimates and Mean VAF

Thirty-three blood samples from patients with solid tumors (one sample per patient) were used to evaluate ctFraction estimates generated by Fragle off-target, Fragle LP-WGS, and ichorCNA. These estimates were compared to OTTER estimates. The ctFraction estimates by the different algorithms and mean VAF (%) in each sample are shown (Supplementary Table S3). The mean VAF (mVAF, %) was calculated as the average VAF of somatic variants detected in each sample by the FCCC ctDNA assay (Supplementary Table S2). Across all comparisons, Spearman (rho) correlation coefficients indicated that the methods preserved the relative ordering of patients by ctDNA levels, despite differences in the underlying analytical approaches.

**Figure 3:**
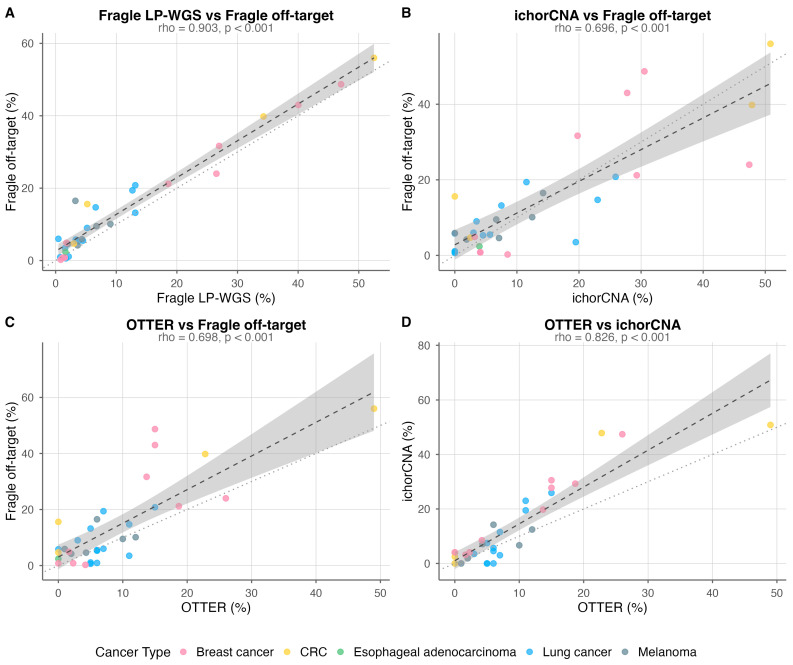
Correlation between ctFraction estimates. (**A**) Fragle off-target vs. Fragle LP-WGS; (**B**) Fragle off-target vs. ichorCNA; (**C**) Fragle off-target vs. OTTER; and (**D**) ichorCNA vs. OTTER. Spearman rho coefficient and p-values are indicated in each panel. The dashed line represents the linear regression fit with the shaded area representing the 95% confidence interval, whereas the dotted gray line is the line of identity (y = x).

Comparative analysis of ctFraction estimation methods demonstrated that Fragle LP-WGS and Fragle off-target had the highest concordance (rho = 0.903, *p* < 0.001), demonstrating highly consistent behavior across samples and supporting the reliability of off-target reads from targeted sequencing as a surrogate for LP-WGS-based estimation (Figure 3A). Comparisons of Fragle off-target with other methods showed moderately strong associations, including ichorCNA (rho = 0.696, *p* < 0.001) (Figure 3B) and OTTER (rho = 0.698, *p* < 0.001) (Figure 3C), reflecting methodological differences between fragmentomic-, CNA-, and mutation-based ctDNA quantification. Additionally, OTTER and ichorCNA exhibited strong and consistent relationships (rho = 0.826, *p* < 0.001), indicating that both methods similarly rank tumor burden across patients, although OTTER incorporates tumor mutations and CNAs, whereas ichorCNA relies exclusively on CNAs (Figure 3D). Correlation analysis demonstrated strong and consistent relationships between ctFraction estimates derived from different algorithms.

**Figure 4:**
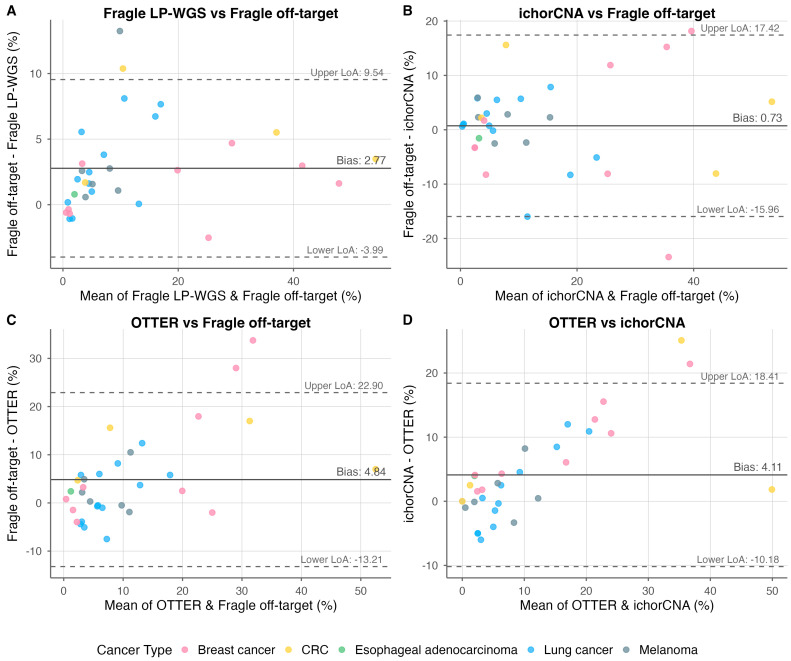
Bland–Altman plots. (**A**) Fragle off-target vs. Fragle LP-WGS; (**B**) Fragle off-target vs. ichorCNA; (**C**) Fragle off-target vs. OTTER; and (**D**) ichorCNA vs. OTTER.

Bland–Altman analysis revealed important differences in agreement between methods. Fragle LP-WGS and Fragle off-target showed the best agreement, with a mean bias of 2.77% and relatively narrow limits of agreement (−3.99% to 9.54%) (Figure 4A). In contrast, comparisons involving other methods demonstrated wider limits of agreement. ichorCNA vs. Fragle off-target showed minimal bias (0.73%) but substantial variability (−15.96% to 17.42%) (Figure 4B), while OTTER vs. Fragle off-target exhibited a positive bias (4.84%) and broad limits of agreement (−13.21% to 22.90%) (Figure 4C). Similarly, ichorCNA vs. OTTER demonstrated a systematic positive bias (4.11%) with wide limits of agreement (−10.18% to 18.41%) (Figure 4D). These findings indicate that while the methods demonstrate strong, consistent relationships, absolute ctFraction estimates differ across approaches, particularly at higher tumor fractions. Consequently, although all methods capture overall tumor burden, they are not directly interchangeable due to fundamental biological and analytical differences among fragmentomic-, CNA-, and mutation-based metrics.

**Figure 5:**
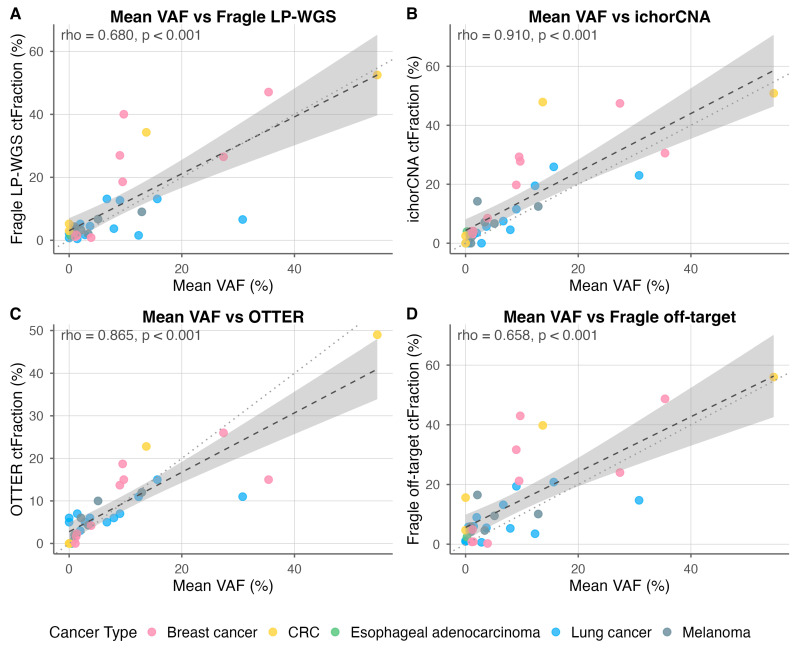
Correlation between mean VAFs (mVAFs) and ctFraction estimates by (**A**) Fragle LP-WGS; (**B**) ichorCNA; (**C**) OTTER; and (**D**) Fragle off-target. Spearman rho correlation coefficients and p-values are indicated in each panel. The dashed line represents the linear regression fit with the shaded area representing the 95% confidence interval, whereas the dotted gray line is the line of identity (y = x).

Correlation analysis was performed to evaluate the relationship between mean variant allele frequency (mVAF) and tumor fraction estimates derived from the different ctFraction algorithms. Mean VAF showed strong and consistent relationships with all ctFraction estimates. The highest correlation was observed for ichorCNA (rho = 0.910, *p* < 0.001), followed by OTTER (rho = 0.865, *p* < 0.001). Fragle-based approaches also demonstrated consistent, though slightly lower correlations, including Fragle LP-WGS (rho = 0.680, *p* < 0.001) and Fragle off-target (rho = 0.658, *p* < 0.001) (Figure 5).

**Figure 6:**
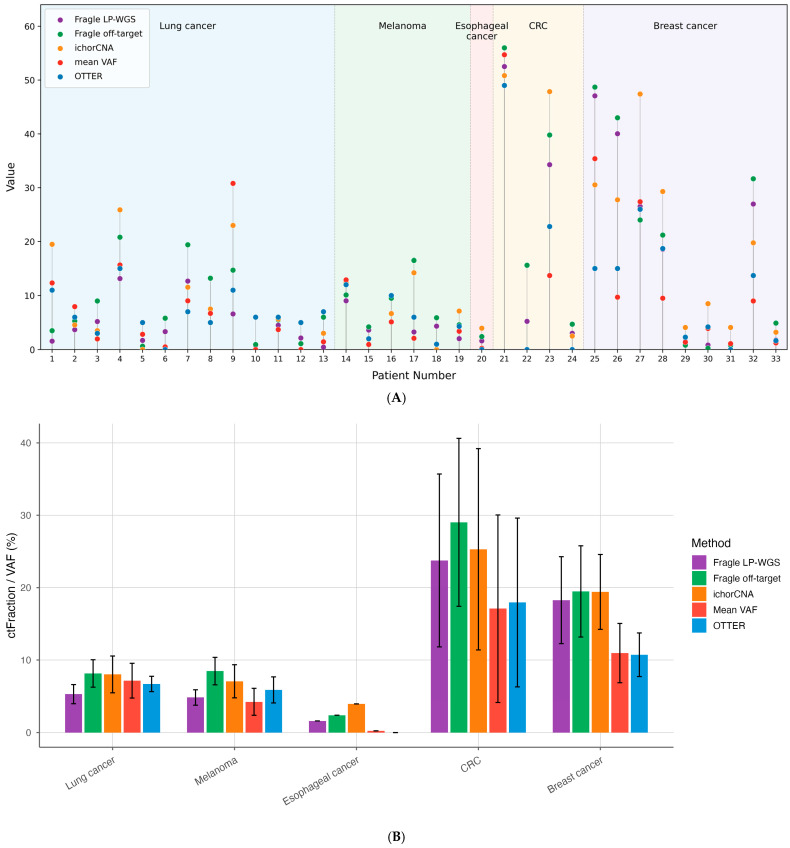
ctFraction estimations by different methods and mean VAF (mVAF) across 33 patients with advanced solid tumors, including lung cancer, melanoma, colon cancer, breast cancer, and esophageal cancer. ctFraction estimates by the different algorithms, together with mVAF, are shown for: (**A**) each patient, and (**B**) each tumor type. Overall, patients with CRC and breast cancer tended to exhibit higher ctFraction and mVAF values than patients with the other tumor types.

These results indicate that ctFraction estimates preserve the relative ordering of ctDNA levels across patients, even when absolute values differ between methods. Across all samples, Fragle off-target returned the highest ctFraction estimates in the majority of cases, while OTTER consistently produced the lowest estimates (Figure 6A and Supplementary Table S3). No variants (mVAF = 0) were detected in Pt.10, Pt.12, Pt.22, and Pt.24 due to low ctFractions (between 0 and 6% across all algorithms), with the exception of Pt.22, for whom Fragle LP-WGS detected a ctFraction of 5.2% and Fragle off-target a ctFraction of 15.6% (Figure 6A). In general, ctFraction estimates and mVAF (%) were higher in patients with colorectal cancer (CRC) and breast cancer than in melanoma, lung, and esophageal cancers (Figure 6B). Pt.21 with colon adenocarcinoma Stage IV, who succumbed to disease 1 year after diagnosis, demonstrated the highest ctFraction and mVAF (Figure 6A).

### 2.3. ctFraction and Variant Allele Frequency Changes During Treatment

In a cohort of 20 patients (Supplementary Table S4), blood samples were collected at baseline (prior to treatment initiation) and after two cycles of therapy (“Post-2”), corresponding to approximately 4–6 weeks of treatment, depending on the therapeutic regimen. The cohort included six patients with lung adenocarcinoma, eight with breast cancer, and six with colorectal cancer (CRC) (Supplementary Table S4).

Plasma ctDNA and matched normal DNA were analyzed for each patient using the FCCC ctDNA assay. A total of seven germline variants, including five germline variants of uncertain significance (VUS), 12 CHIP-associated variants, and 52 somatic variants, were detected in this cohort (Supplementary Tables S1 and S4). Five patients did not show any somatic variants either at baseline or Post-2 (Supplementary Table S4).

ichorCNA and Fragle off-target ctFraction estimates were determined for each patient at baseline and during treatment (Post-2), and the ∆ctFraction was calculated (ctFraction Post-2 − ctFraction Baseline). The ichorCNA and Fragle off-target ΔctFractions and mean ΔVAFs (%) for each patient after two cycles of treatment are shown in Supplementary Table S5. For Pt.10 and Pt.36, the ctFractions were under the LoD for both ichorCNA and Fragle off-target (LoD 3% and LoD 1%, respectively), and therefore ΔctFractions could not be determined.

**Figure 7:**
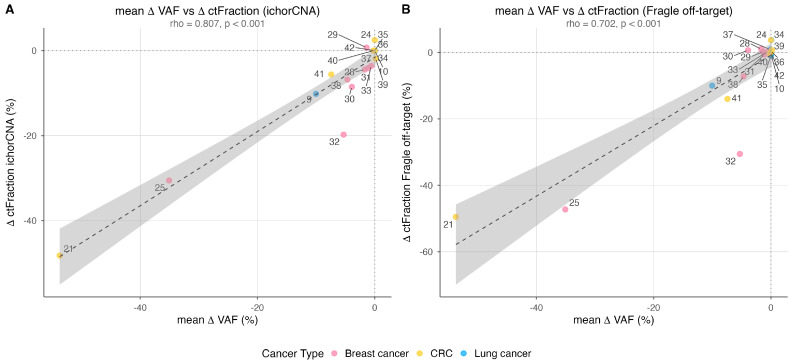
Correlation between mean ∆VAF and ∆ctFraction estimates by (**A**) ichorCNA, and (**B**) Fragle off-target.

ΔctFraction by ichorCNA and Fragle off-target showed a strong positive correlation with mean ∆VAF (rho = 0.807 and rho = 0.702, respectively) (Figure 7). These findings indicate that both copy-number- and fragmentomic-based approaches reliably capture ctDNA dynamics across the cohort, with ichorCNA showing slightly higher agreement with VAF changes. Spearman correlation demonstrated a strong linear relationship between mean ΔVAF and ΔctFraction estimates for both algorithms (Figure 7). Similar results were observed using Pearson correlation, indicating robust associations independent of outlier effects (Pearson r= 0.955 for ΔctFraction by ichorCNA, and r = 0.906 for Δ ctFraction by Fragle off-target).

**Figure 8:**
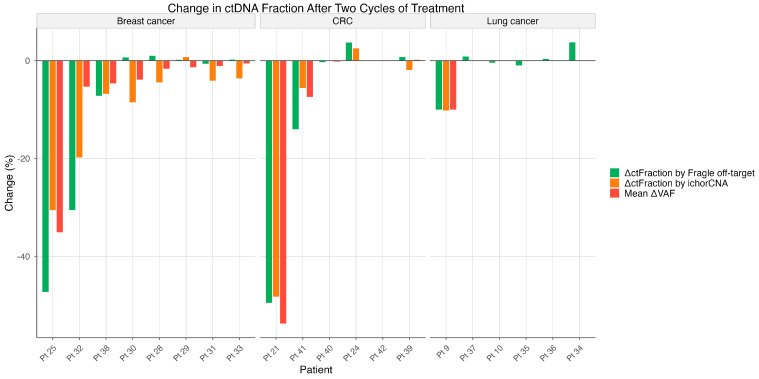
Changes in ctFraction estimates by ichorCNA and Fragle off-target, and change in mean VAF after two cycles of treatment in patients with advanced cancers.

**Figure 9:**
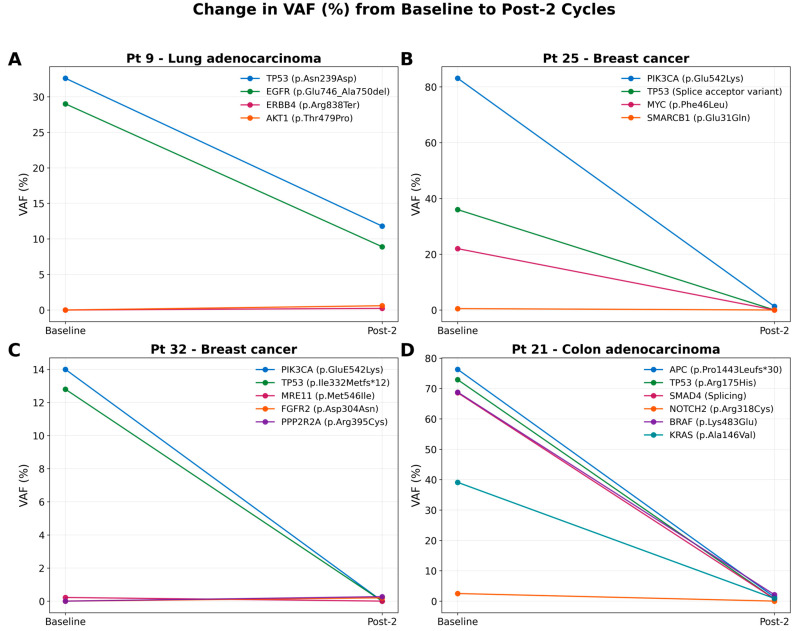
Changes in VAF (%) at baseline and after two cycles of treatment (Post-2) for somatic variants detected in Pt.9, Pt.25, Pt.32, and Pt.21. (**A**) Pt.9 with lung adenocarcinoma treated with osimertinib (targeted therapy for EGFR variant); (**B**) Pt.25 with breast cancer treated with alpelisib and fulvestrant (targeted therapy for HR+/HER2− PIK3CA-mutated disease); (**C**) Pt.32 with breast cancer treated with docetaxel, trastuzumab, and pertuzumab (chemotherapy plus HER2-targeted therapy); and (**D**) Pt.21 with colon adenocarcinoma treated with FOLFOX plus bevacizumab (chemotherapy combined with an anti-VEGF antibody).

Changes in ctDNA burden after two cycles of treatment (Post-2) using Fragle off-target, ichorCNA, and mean VAF measurements in all patients of this cohort (n = 20) are shown in Figure 8. Some patients demonstrated reductions in ctDNA levels across the three methods, consistent with a molecular response to therapy (Figure 8). The overall direction and magnitude of change were generally concordant between approaches, although ΔctFraction by Fragle off-target frequently detected larger decreases in ctDNA burden (Figure 8). Patients 9, 21, 25, and 32 demonstrated the most substantial decreases in both ctFraction estimates (~−10% to −50%) and mean VAF (−5% to −50%) after two cycles of treatment, while the remaining patients showed minimal changes (Figure 8). This molecular response closely paralleled clinical outcomes; radiologic evaluation via CT scans performed closest to baseline, and Post-2 confirmed disease improvement in these same four patients, matching the observed declines in somatic variant allele frequencies and estimated ctFractions (Table 1, Figure 8 and Figure 9).

Additional longitudinal studies with more patients and more time points are necessary to understand how these changes correlate with responses to treatment and imaging.

## 3. Discussion

ctFraction is being increasingly recognized as a clinically relevant pan-cancer biomarker with prognostic value across diverse malignancies [36]. Recent findings indicate that ctFraction may serve as a biomarker for predicting overall survival (OS) across multiple cancer types, including advanced NSCLC, prostate cancer, breast cancer, and colorectal cancer [36]. Several commercially available and laboratory-developed ctDNA assays for tumor genomic profiling now incorporate an estimate of ctFraction because low levels of tumor-derived DNA in plasma may compromise the analytical sensitivity of liquid biopsy testing. Low ctFractions are associated with reduced sensitivity for the detection of somatic variants, particularly CNAs, gene fusions, and low VAF mutations, increasing the risk of false-negative results [35,37].

Despite the widespread clinical adoption of ultra-deep targeted cfDNA sequencing assays for the detection of actionable genomic alterations, most approaches for ctFraction estimation are not compatible with targeted sequencing data as they rely on LP-WGS [24], DNA methylation data [38,39], or require modifications to targeted-panel design [40]. Notably, off-target reads from targeted sequencing, which are typically discarded during standard analysis pipelines, are broadly distributed across the genome and can approximate LP-WGS coverage. Fragle off-target leverages these signals to enable ctDNA quantification directly from targeted sequencing data, eliminating the need for separate LP-WGS and potentially reducing sequencing requirements and workflow complexity [25].

We evaluated ctFraction estimates using three distinct algorithms—Fragle off-target, Fragle LP-WGS, and ichorCNA—in a cohort of 33 patients with advanced solid tumors and compared these results with OTTER (Tempus AI, Inc.) estimates. We found that ctFraction estimates derived from Fragle off-target demonstrated a strong and statistically significant correlation with those obtained from Fragle LP-WGS, in agreement with findings reported by Zhu et al., who employed a different hybrid-capture ctDNA panel [25]. Notably, OTTER generally reported lower ctFraction values and exhibited greater variability across samples compared with both Fragle off-target and ichorCNA. Overall, these findings support Fragle off-target as a stable and reliable primary approach, showing strong concordance with Fragle LP-WGS and more variable agreement with ichorCNA and OTTER. Bland–Altman plots revealed systematic bias between copy-number–based methods (ichorCNA) and fragmentomic-based approaches (Fragle), with ichorCNA tending to underestimate tumor fraction, particularly in low-shedding samples. Our findings are consistent with Finkle et al. [26], who reported a strong correlation between ichorCNA and OTTER. Although they observed only a modest correlation between VAFs and OTTER-derived ctFraction using the Tempus xF assay [26], we identified a strong correlation using VAFs derived from the FCCC ctDNA assay. The difference may be attributable to methodological differences, as our analysis incorporated matched normal DNA to accurately distinguish somatic from germline and CHIP-associated variants, whereas the Tempus approach relies on ctDNA-only sequencing with in silico filtering.

Zhu et al. [25]—who developed the Fragle algorithm—have reported that Fragle has a lower limit of detection (LoD) than ichorCNA; specifically, Fragle can detect and quantify ctDNA at an LoD of 1%, whereas ichorCNA has an LoD of 3% [24,25]. Consistent with these observations, our results demonstrate that Fragle off-target exhibits higher sensitivity compared with ichorCNA, particularly in samples with low tumor burden. In 26 of 62 ctDNA samples analyzed, ichorCNA yielded ctFraction estimates of zero, whereas Fragle off-target detected measurable tumor fractions, underscoring the ability of fragmentomic-based approaches to capture subtle tumor-derived signals that may be missed by copy-number-based approaches. Among a subset of samples with zero ctFraction estimates by ichorCNA, higher values were observed using OTTER, consistent with prior reports demonstrating improved sensitivity of OTTER relative to ichorCNA [26]. Finkle et al. demonstrated that OTTER exhibits higher sensitivity than ichorCNA for ctDNA fraction estimation in low-shedding samples; in 210 samples, ichorCNA estimated tumor fraction as zero while OTTER detected measurable ctDNA fraction, and 60% of these samples also harbored detectable variants by targeted sequencing, supporting the notion that the ichorCNA results represented false negatives [26]. Algorithms such as ichorCNA, which rely on CNAs, may fail to detect ctDNA at low tumor fractions, i.e., <3%, whereas OTTER and fragmentomic-based approaches such as Fragle off-target can identify tumor-derived signals at lower ctDNA levels [25].

Overall, our results demonstrate that while all methods capture ctDNA burden at the cohort level, important differences exist between approaches. Fragle LP-WGS and Fragle off-target showed the highest concordance and agreement, supporting the use of off-target reads from targeted sequencing as a reliable surrogate for LP-WGS-based ctFraction estimation. In contrast, ichorCNA and OTTER, although strongly correlated with each other and with Fragle-based methods, showed greater variability and systematic differences, reflecting their reliance on distinct biological signals, including CNAs and mutation-based estimates.

We observed that ctFraction estimates derived from Fragle, ichorCNA, and OTTER all demonstrated statistically significant positive correlations with mean VAF (mVAF), defined as the average VAF of all detected somatic variants in a sample. Among these methods, ichorCNA and OTTER showed the strongest linear association with mVAF, followed by Fragle LP-WGS and Fragle off-target. We studied the molecular response in a cohort of 20 patients by analyzing ΔctFraction estimates generated by ichorCNA and Fragle off-target, as well as the mean ΔVAF (or Δ mean VAF) after two cycles of treatment. Changes in tumor fraction estimated by both ichorCNA and Fragle off-target were strongly correlated with the mean ΔVAF, supporting concordance between ctFraction estimates and mutation-based metrics. Our results show that both approaches strongly correlate with mutation-derived tumor fraction changes, supporting their use as mutation-independent ctDNA burden metrics.

Notably, Fragle off-target exhibited a consistent positive relationship across the full dynamic range of mVAF values, including at lower tumor fractions where ctDNA detection is most clinically challenging. Importantly, Fragle off-target demonstrated a strong and consistent correlation with mean VAF despite relying exclusively on genome-wide fragmentomic features derived from off-target reads, highlighting the potential of fragmentation-based tumor fraction estimation as an orthogonal, scalable, and mutation-independent approach for ctDNA quantification. Fragle off-target leverages genome-wide cfDNA fragmentation patterns independent of mutation burden. As a result, ctFraction estimates from Fragle off-target provide a more global representation of tumor-derived cfDNA, whereas mVAF reflects only mutation-based signals, partially explaining their only moderate correlation. mVAF is inherently influenced by factors such as the number of detected mutations, tumor heterogeneity, CNAs, and the size and composition of the targeted sequencing panel. Because fragmentation signals are present across the genome, this approach can detect very low levels of ctDNA even in samples with a low mutation burden. However, this benefit may be accompanied by an increased risk of false-positive ctFraction estimates, especially in samples with ctDNA levels close to the limit of detection (LoD). In low-tumor-fraction settings, small variations in cfDNA quality, library complexity, fragment-size distributions, sequencing noise, or stochastic sampling effects may generate low-level ctFraction signals that do not necessarily reflect true tumor-derived DNA. Therefore, ctFraction estimates close to the detection limit should be interpreted with caution. Instead, low-level positive results should be evaluated in the context of orthogonal molecular findings, paired somatic variant data, matched normal analysis, radiographic findings, and longitudinal trends across serial samples. Future studies with larger cohorts, technical replication, and clinical outcome correlation will be needed to better define the specificity, reproducibility, and clinical significance of low-level ctFraction estimates generated by Fragle off-target.

The concept of “molecular response” lacks a universally accepted quantitative definition, and different studies use different thresholds and metrics. Zhang et al. [41] defined molecular response using a ratio of on-treatment to baseline mean VAF, applying a threshold of <0.5 (i.e., ≥50% reduction) to identify responders. Although this method reflects relative change, it may be sensitive to the absolute magnitude of baseline tumor burden and thus be disproportionately influenced by a small number of high-VAF variants. Similarly, in metastatic breast cancer patients treated with CDK4/6 inhibitors and endocrine therapy, a mean VAF ratio < 0.3 after one cycle of treatment was associated with improved PFS, further supporting the clinical relevance of ctDNA-based molecular response assessment [42]. In the present study, we employed a sum-of-differences approach that computed mean ΔVAF as the mean across all detected somatic variants of the per-variant difference between on-treatment and baseline VAF. This approach weights each somatic variant equally and thereby reduces the risk that a single mutation disproportionately drives the aggregate response estimate.

ctFraction has been increasingly utilized to evaluate molecular response in clinical trials. A recent study in advanced breast cancer demonstrated the improvement of risk stratification in HR-positive/HER2-negative patients when baseline ctDNA-derived ctFraction estimations by ichorCNA were integrated with other clinical variables [43]. In the phase II TRIUMPH trial, an early decrease in ctFraction at 3 weeks following treatment with pertuzumab plus trastuzumab in HER2-amplified metastatic colorectal cancer patients was associated with improved PFS and OS [43]. Consistent with these findings, longitudinal analyses using Fragle ctFraction estimates have demonstrated strong concordance between ctDNA dynamics and treatment response across multiple cancer types [25]. Zhu et al. showed that ctFraction changes in CRC patients measured by Fragle closely paralleled radiographic responses, while in resected lung cancer, Fragle outperformed tumor-naïve gene panels in predicting minimal residual disease (MRD), highlighting its superior sensitivity for risk stratification [25]. Overall, Fragle-based ctDNA quantification has shown strong correlation with treatment response assessed by imaging modalities, underscoring the clinical value of fragmentomic-based approaches for longitudinal disease monitoring. Consistent with prior evidence, our analysis of 20 patients with advanced solid tumors showed that a subset exhibited concordant decreases in ctFraction estimates by ichorCNA and Fragle off-target and mean ΔVAF after two cycles of therapy. Notably, Pt.21 and Pt.25 demonstrated approximately 50% reductions in both metrics, consistent with a molecular response. Importantly, in the four patients with the most pronounced molecular reductions, concordant radiological improvement was documented, providing preliminary evidence that mean ΔVAF and ΔctFraction signals capture clinically meaningful treatment response even within this small dataset. Although in our study the longitudinal cohort (n = 20) was limited by a small sample size, few time points, and predominantly low baseline ctFraction levels, a subset of patients with detectable ctDNA demonstrated concordant changes in ctFraction and VAF that paralleled clinical response. Specifically, only 4 of 20 patients showed measurable ctFraction changes between baseline and Post-2 that were consistent with treatment response. In the remaining patients, ctFraction levels were very low or undetectable at baseline and remained low after two cycles of treatment (Post-2), limiting the ability to assess temporal changes. These findings suggest that ctFraction may be useful for monitoring dynamic changes in ctDNA levels in patients with measurable circulating disease; however, definitive conclusions regarding response monitoring need larger prospective studies.

An additional limitation of this study is that technical replicates were not performed for samples with ctFraction estimates near or below the reported limits of detection of the evaluated methods. In low-ctDNA or low-biomass samples, stochastic sampling effects, random molecular dropout, and amplification variability may influence ctFraction estimates, particularly when tumor-derived DNA represents only a very small fraction of total cfDNA. Therefore, ctFraction values close to the limit of detection should be interpreted with caution, especially when used to assess small changes over time. Future studies incorporating technical replicates, orthogonal confirmation, or larger longitudinal cohorts will be important to better define the reproducibility and clinical reliability of ctFraction estimation in low-tumor-fraction settings. In patients with stage III colon cancer, treatment decisions remain challenging, and many patients are potentially overtreated despite having MRD after surgery. A substantial proportion of these patients may already be cured by surgery alone yet still receive adjuvant chemotherapy associated with significant toxicity and long-term side effects [44]. In this setting, ctDNA assays and ctFraction analysis are promising tools for identifying individuals with persistent molecular disease who remain at higher risk of recurrence.

The integration of ctFraction and VAF dynamics enabled sensitive detection of early molecular responses to therapy, highlighting the complementary value of fragmentomic- and mutation-based approaches for monitoring treatment efficacy. Importantly, Fragle off-target enables ctFraction estimation directly from targeted sequencing data without requiring additional low-pass whole-genome sequencing, potentially reducing costs, sample requirements, and laboratory workflow complexity. Collectively, these findings support the incorporation of fragmentomic-based methodologies into ctDNA analysis workflows to enhance early response assessment and longitudinal disease monitoring in precision oncology. Furthermore, ctFraction estimation may serve as a complementary biomarker in ctDNA tumor profiling assays by identifying samples with low tumor shedding and potentially reduced assay sensitivity, thereby improving interpretation of negative or low-variant findings. Larger prospective, multicenter studies, together with formal analytical performance evaluations, are required to establish the clinical validity, reproducibility, and generalizability of Fragle off-target across diverse patient populations and cancer types before it can be adopted for routine clinical use or to inform treatment decisions.

## 4. Materials and Methods

### 4.1. Sample Collection and Patient Characteristics

Blood samples used in this study were from patients with advanced solid tumors treated at Fox Chase Cancer Center, Philadelphia, PA, USA. For this study, patients provided written informed consent under an IRB-approved research protocol (IRB 19-9030). A total of 42 patients with several tumor types such as NSCLC, breast cancer, melanoma, CRC, and esophageal cancer were included in the study (Supplementary Table S1). In the cohort of 33 patients, one sample from each patient was studied (Figure 1A). For the cohort of 20 patients, blood samples were studied at baseline and after two cycles of treatment (Post-2; around 4–6 weeks after treatment initiation depending on the drug) (Figure 1B). Patients included in the longitudinal cohort were selected based on the availability of paired baseline and Post-2 plasma samples with sufficient cfDNA input for library preparation, within the constraints of available study resources. The cohort was enriched for tumor types most frequently submitted for molecular analysis at our institution, including lung cancer, breast cancer, and colorectal cancer. Patients received different systemic therapies at various lines of treatment, and inclusion was not restricted to a specific drug, treatment regimen, line of therapy, or clinical response. Some patients were excluded from the study either because they died before the post-treatment time point, blood was drawn at only one time point, or there was insufficient blood volume. The longitudinal cohort (n = 20) included 11 patients who were also part of the initial 33 patient cohort: Pt.9, Pt.10, Pt.21, Pt.24, Pt.25, Pt.28, Pt.29, Pt.30, Pt.31, Pt.32, and Pt.33. Patients’ demographics were collected (Supplementary Table S1).

### 4.2. Imaging Analysis

Treatment efficacy was evaluated using conventional radiological assessments (CT or MRI) every 12 weeks, applying RECIST 1.1 (Response Evaluation Criteria in Solid Tumors) criteria. Reported results are derived from clinical radiology reports and reviewed against RECIST. RECIST classifies response based on CT/MRI imaging, with one-dimensional measurements from CT/MRI scans (baseline vs. follow-up). It classifies response into Complete Response (CR), Partial Response (PR), Stable Disease (SD), or Progressive Disease (PD) using a maximum of five target lesions (max. two per organ). CR implies the disappearance of all target lesions and pathological lymph nodes; PR, at least 30% decrease; PD, at least 20% increase in target lesions; and SD, neither sufficient shrinkage to quantify for PR nor sufficient increase to quantify for PD. Imaging was evaluated at two time points, near-baseline and Post-2, although for most patients these times were not exactly matched to the blood draw timepoints for ctDNA studies due to the retrospective nature of the study.

### 4.3. Blood Processing, Plasma cfDNA, and Genomic DNA (gDNA) Extractions

Whole blood from each patient was collected in two cell-free DNA BCT tubes (STRECK, La Vista, NE, USA) (10 mL each tube; ~20 mL total) and centrifuged at 1900× *g* for 15 min (room temperature) to separate the plasma supernatant from red blood cells (RBCs). The buffy coat and lower layer with RBCs were aliquoted and frozen at −80 °C to later prepare genomic DNA (gDNA). The plasma supernatant was further centrifuged in a high-speed micro-centrifuge at 16,000× *g* for 15 min at 4 °C and the supernatant was aliquoted and frozen at −80 °C until cfDNA extraction. Extraction of cfDNA was performed using the QIAamp MinElute ccfDNA Midi kit (Cat# 55284) (Qiagen, Germantown, MD, USA) according to the manufacturer’s instructions. Finally, the cfDNA was eluted from the Qiagen columns in 60–70 µL and the double strand cfDNA concentration was measured in the Qubit 4 fluorometer (Invitrogen, Waltham, MA, USA) using the Qubit^TM^ 1X dsDNA HS assay kit (Cat# Q33231) (Invitrogen, Waltham, MA, USA). gDNA from white blood cells (WBCs) was extracted using the QIAamp DNA Blood Mini kit (Qiagen, Germantown, MD, USA).

### 4.4. FCCC ctDNA Assay: Library Preparation and Sequence Analysis

Genetic variants in plasma ctDNA and matched gDNA samples were studied using the validated FCCC ctDNA assay [34], a targeted hybrid-capture assay designed to identify variants across the full coding regions of 150 genes, covering approximately 0.645 Mb of genomic content. The assay detects single-nucleotide variants (SNVs) and small insertions/deletions (indels). The genes included in the 150-gene panel are listed in Supplementary Table S6. Of these, 84 genes (80%) overlap with the Tempus xF panel, which was used as part of the analytical validation of the FCCC ctDNA assay [34]. Libraries were prepared using 40 ng of cfDNA following the Illumina cfDNA Prep with Enrichment kits (Cat# 20104105, Cat# 20104107, and Cat# 20034701) (Illumina, San Diego, CA, USA). For matched normal DNA (gDNA), libraries were prepared using 40 ng of gDNA previously fragmented in 150–200 bp using Agilent’s Sure Enzymatic Fragmentation kit (Cat# 5191-4080) (Agilent Technologies, Santa Clara, CA, USA). The workflow for library preparations includes unique molecular identifiers (UMIs) for error correction and reduction of false positives, enabling accurate and sensitive detection of low-frequency mutations. Pre-capture libraries were prepared following the manufacturer’s instructions and resuspended in a final volume of 45 µL. A total of 7.5 µL of the pre-capture library was then used to hybridize to the capture 150-gene panel, and the remaining 37.5 µL of the pre-capture library was kept at −80 °C to be used later in LP-WGS, as described below. The custom 150 gene panel (IDT, Coralville, IA, USA) consisting of 5389 single-stranded DNA probes was used to enrich the libraries (Supplementary Table S6). Libraries were quantified using the TapeStation system (Agilent Technologies, CA, USA), and 10 enriched libraries were pooled and sequenced using 2 × 150 bp paired end reads with ten-base indexing on the NextSeq 2000 using a P4 XLEAP-SBS cartridge (Illumina, San Diego, CA, USA). Ultra-deep sequencing, also known as deep coverage or high-depth sequencing, was performed for tumor profiling. Plasma ctDNA and matched gDNA for all the samples were sequenced at a coverage of ~4200× (post-deduplication).

Once sequencing was completed, the data were automatically transferred to Illumina BaseSpace for demultiplexing and FASTQ generation using BCL Convert. Secondary analysis was performed using DRAGEN ILMN cfDNA Prep with Enrichment App v.4.0.3 (Illumina, San Diego, CA, USA) in BaseSpace^TM^ Sequence Hub. Variant allele frequencies (VAFs) from VCF files were determined by calculating the ratio of sequencing reads supporting the variant allele versus the total (mutant + wild type) number of reads at a given locus.

VCF and BAM files were then uploaded to VarSeq Suite (Golden Helix, Bozeman, MT, USA) for tertiary analysis. Using VarSeq, variant filtering was performed using a standardized multi-step filter chain designed to identify high-confidence somatic alterations while minimizing sequencing artifacts. Initial quality filters included assessment of read depth, VAF, base quality, strand bias, and other assay-specific quality metrics, and only variants meeting predefined validation criteria were retained for further review. Variants were annotated using population frequency databases, functional consequence annotations, and cancer-specific knowledge bases. Common polymorphisms were excluded based on population allele frequency thresholds. A matched normal sample (gDNA) was analyzed concurrently with each plasma ctDNA sample to facilitate the identification of germline and CHIP variants and distinguish them from somatic alterations. Using VarSeq, variants were processed through three parallel filter chains: (1) somatic variants (detected in ctDNA and absent from the matched normal sample); (2) clinically relevant germline variants (detected in both ctDNA and paired normal gDNA and exhibiting a VAF ≥40% in the matched normal sample); and (3) CHIP variants (detected in both ctDNA and paired normal samples) in genes commonly implicated in clonal hematopoiesis, including *DNMT3A*, *TET2*, *TERT*, *ASXL1*, *PPM1D*, *TP53*, *JAK2*, *PTPN11*, *SF3B1*, *SRSF2*, *KIT*, and *ATM* [34]. The analytical cutoffs established for the FCCC ctDNA assay were 0.2% VAF for SNV and 0.4% VAF for indels [34]. Following these automated steps, all variants underwent manual review using an integrated genome visualization tool to confirm sequence quality, and relevant variants were manually selected for clinical interpretation. Classification of the variants were performed using VSClinical and Cancer KB (Golden Helix, MT, USA) following AMP guidelines for somatic variants and ACMG guidelines for germline variants.

The final sets of high-confidence somatic variants (Supplementary Table S2 for the 33-patient cohort; Supplementary Table S4 for the 20-patient longitudinal cohort) were used to calculate the mean VAF and to assess its correlation with ctFraction estimates generated by ichorCNA, Fragle LP-WGS, Fragle off-target, and OTTER.

For the longitudinal cohort of 20 patients, variants detected in both baseline and on-treatment (Post-2) samples were retained for longitudinal analysis, even if the VAF in one sample was below the assay reporting threshold (<0.2% for SNVs or <0.4% for indels), provided that the same variant was detected in the paired sample at or above the corresponding threshold (≥0.2% for SNVs or ≥0.4% for indels). If a variant was detected only at baseline, its VAF in the on-treatment sample was assigned a value of 0; conversely, if a variant was detected only in the on-treatment sample, its baseline VAF was assigned a value of 0.

Fragle off-target ctFraction estimates were generated using off-target reads from the deep targeted sequencing data of the FCCC ctDNA assay, whereas ctFraction estimates by ichorCNA and Fragle LP-WGS were generated from separate low-pass whole-genome sequencing (0.5×–1×) libraries prepared prior to hybridization with the 150-gene panel.

### 4.5. Low-Pass Whole-Genome Sequencing (LP-WGS)

LP-WGS, also known as shallow whole-genome sequencing, of plasma cfDNA was performed to calculate ctFractions by ichorCNA and Fragle LP-WGS. The 37.5 µL pre-capture libraries which were kept at −80 °C, as previously described in Section 4.4, were used. Briefly, the whole-genome libraries were cleaned using Illumina purification beads (IPBs) and resuspended in 35 µL RSB buffer (Illumina, San Diego, CA, USA). These libraries were amplified by two PCR cycles (98 °C for 10 s; 60 °C for 30 s and 72 °C for 30 s), cleaned with IPBs, and resuspended in 50 µL RSB buffer following the manufacturer’s instructions (Illumina, San Diego, CA, USA). Twelve whole-genome libraries were pooled together and sequenced in NextSeq 2000 using a P1 XLEAP-SBS cartridge at 0.5×–1× coverage (LP-WGS).

### 4.6. Algorithms to Calculate ctFraction

#### 4.6.1. ichorCNA Algorithm

The ichorCNA algorithm is based on the detection of broad CNA across the genome [24]. Chromosomal copy number changes occur frequently in human cancers. The ichorCNA algorithm requires whole-genome sequencing (WGS) data, and it is not optimal for targeted panels. It quantifies tumor content in ctDNA using LP-WGS data and the ctFraction is calculated by inference through fitting coverage shifts to reference diploid genomes. ichorCNA has not been validated in clinical settings [24]. ichorCNA was used according to usage guidelines and the default parameters to compute read count coverage with the Hidden Markov Model (HMM) copy Suite, followed by deducing tumor fraction with the ichorCNA R package (version 0.3.2). The LoD for ichorCNA has been previously established as 3% [24].

#### 4.6.2. FrAGLe (Fragment Length and Genome-Wide Signal) Algorithm

Fragle estimates ctDNA levels using fragmentomic, specifically by modeling the density distribution of cfDNA fragment lengths and quantifying the enrichment of shorter tumor-derived fragments relative to background cfDNA. The approach can be applied to both LP-WGS data (Fragle LP-WGS) and off-target reads obtained from deep targeted sequencing (Fragle off-target) [25]. Fragle LP-WGS uses sequencing data from shallow sequencing; meanwhile, Fragle off-target uses sequencing data from off-target reads generated during deep targeted hybrid-capture sequencing [25]. Fragle software was applied to LP-WGS and off-target BAM files aligned to the hg19 reference genome without any preprocessing, as recommended [25]. The LoD for Fragle has been established as 1% [25]. Fragle has not been validated in clinical settings [25]. The Fragle software (version 1.1) was developed by Zhu et al. [25] and is available at https://github.com/skandlab/FRAGLE (accessed on 6 May 2025).

#### 4.6.3. OTTER Algorithm

OTTER is a proprietary, clinically validated algorithm developed by Tempus AI (Chicago, IL, USA) that estimates ctDNA fraction directly from targeted-panel sequencing data generated by assays such as Tempus xF [26,45,46]. OTTER generates ctFraction estimates from single targeted-panel sequencing experiments and does not require additional sequencing [26]. This ensemble algorithm integrates signals from somatic variants (SNVs and indels), CNVs, and B-allele frequencies (BAFs) with background error modeling.

cfDNA from a total of 33 patients (Supplementary Table S1, Pt.1–33) were sent to Tempus for tumor profiling by the xF assay as part of the validation of the FCCC ctDNA assay [34]. The xF assay detects SNVs and indels in 105 genes, copy number gains in six genes, and gene rearrangements in seven genes, spanning approximately 0.3 Mb of genomic space; a total of 84 genes from the xF assay overlapped with the FCCC 150-gene ctDNA panel (80% overlap) [34]. Tempus also provided OTTER (v0.3) ctFraction estimates for each sample, which were used as a research approach to evaluate the potential utility of other algorithms (Fragle and ichorCNA).

### 4.7. Molecular Response Using VAFs

∆VAF was calculated per patient as the sum of the on-treatment VAF minus the baseline VAF for each detected somatic variant divided by the number of detected variants [41]. Delta-VAF was calculated as ∆VAF_j_ = $\sum_{i=1}^{n}$ (on-treatment VAF_i,j_ − baseline VAF_i,j_)/n. In this equation, on-treatment VAF represents the on-treatment VAF for somatic variant i in patient j, baseline VAF for somatic variant i in patient j, and n is the number of somatic variants at either the baseline or on-treatment time point in patient j. ∆VAF < 0 reflects a decline in mean ctDNA level.

Different methods to calculate molecular response are available [47]; for example, Zhang et al. [41] used the ratio of the mean VAF on-treatment to the baseline and a cutoff of 50% to define molecular responders (<50%) or molecular non-responders (≥50%).

### 4.8. Statistics

Statistical analyses were performed to evaluate the relationships between ctFraction estimates derived from different algorithms and mean variant allele frequency (mean VAF). Correlations between methods were assessed using Spearman’s rank correlation coefficient (rho). Spearman correlation was prioritized to assess consistency in the ranking of ctFraction estimates across patients, given the non-normal distribution and wide dynamic range of ctDNA measurements. Statistical significance was determined using two-sided *p*-values. Agreement between ctFraction estimation methods was evaluated using Bland–Altman analysis, including calculation of mean differences (bias) and 95% limits of agreement (mean difference ± 1.96 standard deviations). These analyses were used to assess whether methods could be used interchangeably at the individual sample level. Pearson correlation analysis was used to evaluate the linear relationship between longitudinal changes in mean variant allele frequency (mean ΔVAF) and changes in ctFraction estimates (ΔctFraction) derived from different algorithms. Pearson correlation coefficients (r) and two-sided *p*-values were calculated to assess the strength and significance of these associations. Pearson correlation was selected because the analyses aimed to evaluate proportional changes between continuous variables across serial patient samples. All statistical analyses were performed using R (version 4.4.0; R Foundation for Statistical Computing, Vienna, Austria) and Python (version 3.12.2; Python Software Foundation, Wilmington, DE, USA) with standard scientific libraries.

## 5. Conclusions

ctFraction estimates derived from Fragle demonstrated strong concordance with established ctDNA quantification approaches, including ichorCNA and OTTER, across a diverse cohort of patients with advanced solid tumors. Despite differences in the underlying analytical methodologies, all approaches showed consistent patient stratification according to ctDNA levels, supporting the robustness of ctFraction as a biomarker of circulating tumor DNA abundance. While the methods demonstrate strong, consistent relationships, absolute ctFraction estimates differ across approaches, particularly at higher tumor fractions. Consequently, although all methods capture overall tumor burden, they are not directly interchangeable due to fundamental biological and analytical differences among fragmentomic-, CNA-, and mutation-based metrics. ctFraction estimates preserve the relative ordering of ctDNA levels across patients, even when absolute values differ between methods.

The main potential advantage of Fragle off-target in clinical settings is that it does not require additional LP-WGS or separate sequencing libraries, which could reduce sequencing costs and workflow complexity. Because the approach uses data generated from targeted sequencing assays, ctFraction estimation could theoretically be integrated into existing ctDNA workflows without requiring an additional assay, thereby potentially reducing turnaround time compared with workflows that require separate LP-WGS. The potential clinical value of Fragle off-target lies in its ability to provide a cost-effective, mutation-independent ctFraction estimate directly from existing targeted sequencing data, with possible applications in longitudinal patient monitoring. However, its use for treatment decisions will require further validation in larger prospective studies.

Longitudinal analysis was limited by low baseline ctFraction levels in most patients; however, patients with measurable ctDNA showed ctFraction changes that paralleled VAF dynamics and clinical response, supporting further investigation in larger cohorts. Larger prospective, multicenter studies and formal analytical performance assessments are required to establish the clinical validity, reproducibility, and generalizability of Fragle off-target across diverse patient populations and cancer types before this approach can be considered for routine clinical use or treatment decision-making.

## Figures and Tables

**Table 1:** Radiologic evaluation in a cohort of 20 patients with advanced solid tumors. The timing (in months) of blood collection and imaging studies are indicated. In the Blood Dates column, the baseline blood draw is designated as Month 0. In the CT Dates column, the CT scans performed closest to the baseline and post-cycle 2 (Post-2) blood collection time points are reported relative to the baseline blood draw (Month 0). For Patient 31, “0/?” in the CT Dates column indicates that no follow-up CT scan was available for comparison with the baseline CT scan performed at Month 0. For Pt.30, PET imaging was used in place of CT scans.

Pt ID	Type of Tumor	Blood Dates	CT Dates	CT Scan Evaluation
		Baseline/Post-2	Before treat./After treat.	
		Month #	Month #	
9	Lung adenoc.	0/3	−1/2	Improved, treatment response
10	Lung adenoc.	0/2.5	1/3	Stable disease (SD)
34	Lung adenoc.	0/2.8	1.8/2.8	Improved, treatment response
35	Lung adenoc.	0/1.4	0/1.4	Improved, mass is better
36	Lung adenoc.	0/2.3	0/2.3	Worsened with possible infection
37	Lung adenoc.	0/0.7	−2.8/0.7	Stable
25	Breast cancer	0/1	0/3	Stable-to-improved
28	Breast cancer	0/2	0/2.4	Worsened
29	Breast cancer	0/0.9	0/3.6	Improved, near-resolved
30	Breast cancer	0/2.1	0/3.6 (PET)	Better resolution (PET scan)
31	Breast cancer	0/1.4	0/?	No follow-up CT available to compare
32	Breast cancer	0/1.5	0/5.8	Stable-to-improved
33	Breast cancer	0/1.4	0/8	Worsened, with necrosis, progressed
38	Breast cancer	0/1.8	0/2.2	Slightly worse
21	Colon adenoc.	0/1.4	−1/1.4	Overall better, response
24	Colon adenoc.	0/1.4	0/3.8	Stable
39	Rectal adenoc.	0/0.9	−1/4.4	Slightly worsened initially, with subsequent improvement
40	Rectal adenoc.	0/0.5	0/2.5	Stable liver, nodes better
41	Colon adenoc.	0/1	0/2.5	Stable, overall same
42	Colon adenoc.	0/1.8	0/4.4	Stable, with subsequent improvement (slightly better)

## Data Availability

The original contributions presented in this study are included in the article/Supplementary Materials. Further inquiries can be directed to the corresponding author.

## Supplementary Materials

The following supporting information can be downloaded at: https://www.mdpi.com/article/10.3390/ijms27136078/s1.

## Abbreviations

cfDNA, cell-free DNA; CHIP, clonal hematopoiesis of indeterminate potential; CT scan, computed tomography scan; ctDNA, circulating tumor DNA; ctFraction, circulating tumor DNA fraction; CNA, copy number alterations; CRC, colorectal cancer; gDNA, genomic DNA; Fragle, Fragment Length and Genome-wide signal; indels, small insertions and deletions; LP-WGS, low-pass whole-genome sequencing; mVAF, mean VAF; MRD, minimal residual disease; NGS, next-generation sequencing; OTTER, Off-Target Tumor Estimation Routine; Post-2, after two cycles of treatment; RBCs, red blood cells; r, Pearson correlation coefficient; rho, Spearman correlation coefficient; SNVs, single nucleotide variants; TKIs, tyrosine kinase inhibitors; VAF, variant allele frequency; mVAF, mean VAF; VUS, variant of uncertain significance; WBCs, white blood cells; WGS, whole-genome sequencing.
