# Supplementary material for: Off-Target-Based Tumor Fraction Estimation from Targeted Sequencing Shows Concordance with Orthogonal Methods Across Advanced Solid Tumors
Source: Int J Mol Sci. 2026 Jul 7;27(13):6078. doi: 10.3390/ijms27136078 (PMC13362176; doi:10.3390/ijms27136078)
Supplement: Supplementary file 1 [file ijms-27-06078-s001.zip › ijms-4366076-supplementary.pdf]

Patient ID	Type of cancer	Stage	Sex	Age at diagnosis	Germline (% VAF)	CHIPs (%VAF)
1	Lung adenocarcinoma	IV	M	63		
2	Lung adenocarcinoma	IV	M	73		
3	Lung squamous cell carcinoma	IV	M	75		TET2 c.3594+1 G>A (4.1%)-Oncogenic
						TP53 Arg273His (0.9%)- Oncogenic
						KIT Pro166Arg (0.61%)- VUS
4	Lung adenocarcinoma	IV	M	28	ROS1 Arg2096Trp (31% in ctDNA; 48% in normal DNA)- VUS	
5	Lung squamous cell carcinoma	IV	F	44	MUTYH Gly368Asp (49.3%)- Pathogenic	
					MAP2K2 His243Asp (48%)- VUS	
					ATR Arg273His (47%)-VUS	
6	Lung adenocarcinoma	III	F	80		
7	Lung adenocarcinoma	IV	F	67		
8	Lung adenocarcinoma	IV	M	78 (prostate cancer histoty)		
9	Lung adenocarcinoma	IV	M	54		
10	Lung adenocarcinoma	IV	F	80	ATM Leu3034Trp (49%)- VUS	DNMT3A Arg88His (41%)- Oncogenic
						NF1 Arg2226Ter (0.37%)- Likely Oncog.
11	Lung adenocarcinoma	IV	M	70	ATM Arg447* (59%)-Pathogenic	DNMT3A Ile780Thr (2.5%)- Likely Oncog.
12	Lung adenocarcinoma	IV	M	63		ATM Leu2722Arg (6.2%)- Likely Oncog.
13	Lung squamous cell carcinoma	IV	F	65	JAK3 Val487Met (48%)- VUS	
14	Melanoma	IV	F	46	ALK Ala1200Val (41%)- VUS	
15	Melanoma	IV	M	62		
16	Melanoma	IV	F	63		
17	Melanoma	IV	M	84		
18	Melanoma	IV	M	86		TP53 Asn239Asp (39%)- Oncogenic
						DNMT3A Val636Met (40.2%)- Likely Oncog.
19	Melanoma	IV	F	57 (breast cancer history)		DNMT3A Lys841Ter (10%)- Oncogenic
20	Esophageal adenocarcinoma	IV	F	46	MYC Arg436Gln (50%)- VUS	
					FLT3 Asn306His (49%)- VUS	
21	Colon adenocarcinoma	IV	F	69	PTPN11 p.Asn58Ser (15% in ctDNA; 47% in gDNA)- VUS	
22	Colon adenocarcinoma	III	F	64	MLH1 Leu607His (49%)- VUS	
23	Rectal adenocarcinoma	IV	M	82		ATR Asn1022Ser (0.74%)- VUS
24	Colon adenocarcinoma	III	F	58		TET2 Arg1261Cys (5.4%)- Likely Oncog.
						DNMT3A c.1123-1G>A (0.46%)- VUS
						DNMT3A Gly298Val (0.27%)- VUS
25	Breast cancer	IV	F	44	TSC1 Gln1054Arg (43%)- VUS	
26	Breast cancer	IV	F	60		
27	Breast cancer	IV	F	51	RET Val648Ile (49.7%)- VUS	TP53 Cys176Tyr (3.7%)- Oncogenic
28	Breast cancer	IV	F	63		TET2 Tyr899Ilefs*22 (0.72%)- Likely Oncog.
29	Breast cancer	III	F	35	BRCA1 Ser1253Argfs* (53%)- Pathog.	
					PTPN11 Asn58Ser (51% in ctDNA; 46% in gDNA)- VUS	
30	Breast cancer	IV	F	57 (lung cancer history)	MUTYH Tyr179Cys (48%)- Likely Pathog.	
31	Breast cancer (IBC)	III	F	57		
32	Breast cancer	IV	F	43	MET Ile565Ser (45%)- VUS	
33	Breast cancer	IV	F	50		TP53 Cys135Gly (0.51%)- Oncogenic
34	Lung adenocarcinoma	III	M	68		
35	Lung adenocarcinoma	IV	F	65		
36	Lung adenocarcinoma	IV	M	73		
37	Lung adenocarcinoma	IV	F	73		
38	Breast cancer	IV	F	61		TP53 Arg273His (0.4%)- Oncogenic
						TP53 Thr125Met (0.38%)- Oncogenic
39	Rectal cancer	IV	M	52		ATM Val410Cysfs*10 (2%)- Likely Oncogenic
40	Rectal cancer	IV	F	63		
41	Colon adenocarcinoma	IV	M	70		
42	Colon adenocarcinoma	III	F	68 (breast cancer history)		TP53 Gly245Ser (0.41%)- Oncogenic
						TET2 Lys119Arg (0.2%)-VUS

Supplementary Table S1. Patient demographics. Type of cancer, stage of the disease at the time of the blood drawn for molecular studies, sex and age at diagnosis are indicated. Germline mutations and CHIP variants are shown. IBC, inflammatory breast cancer; VUS, variant of uncertain significance.

Suppl. Table S2.

Patient ID	Type of cancer	Gene	DNA Variant	Amino Acid change	150 genes assay VAF (%)	Mean VAF (%)	Type of variant
1	Lung adenocarcinoma	**TP53**	c.818G>A	p.Arg273His	**0.27**	**12.33**	**Oncogenic**
		**CDKN2A**	c.301G>T	p.Gly101Trp	**17**		**Oncogenic**
		**TP53**	c.313G>T	p.Gly105Cys	**22.5**		**Oncogenic**
		**NF1**	c.7127-1G>A	Splicing	**18.4**		**Oncogenic**
		**STK11**	c.921-2A>C	Splicing	**0.53**		**Likely Oncogenic**
		**RB1**	c.264+5G>A	Splicing	**18.7**		**Likely Oncogenic**
		**KEAP1**	c.163_168delACCCTG	p.Thr55_Leu56del	**21.6**		**VUS**
		**MET**	c.3583C>A	p.Leu1195Ile	**6.3**		**VUS**
		**MPL**	c.1382T>A	p.Leu461Gln	**1.7**		**VUS**
		**ERBB4**	c.3280delG	p.A1094Hfs*58	**16.3**		**VUS**
2	Lung adenocarcinoma	**KRAS**	c.35G>C	p.Gly12Ala	**24.8**	**7.94**	**Tier 1**
		**TP53**	c.743G>A	p.Arg248Gln	**5.6**		**Oncogenic**
		**ALK**	c.3885G>A	p.Trp1295Ter	**3.6**		**VUS**
		**ALK**	c.3931G>C	p.Asp1311His	**3.3**		**VUS**
		**JAK3**	c.530A>T	p.Gln177Leu	**2.4**		**VUS**
3	Lung squamous cell carcinoma	**TP53**	c.659A>G	p.Tyr220Cys	**2.2**	**1.97**	**Oncogenic**
		**PTEN**	c.72C>A	p.Asp24Glu	**0.32**		**Oncogenic**
		**MSH3**	c.1148delA	p.Lys383Argfs*32	**0.44**		**Oncogenic**
		**NF1**	c.6974_6977delATAG	p.Asp2325Valfs*49	**1.8**		**Oncogenic**
		**NOTCH1**	c.141-1G>T	Splice acceptoring	**2**		**VUS**
		**KDR**	c.3173A>G	p.Asp1058Gly	**2.9**		**VUS**
		**SRC**	c.448G>A	p.Glu150Lys	**2.4**		**VUS**
		**BRIP1**	c.3440dupA	p.Asn1147Lysfs*2	**0.77**		**VUS**
		**FGF10**	c.121G>A	p.Gly41Ser	**4.9**		**VUS**
4	Lung adenocarcinoma	**SMARCA4**	c.305delG	p.Gly102Alafs*201	**44.3**	**15.65**	**Likely Oncogenic**
		**CDH1**	c.817G>A	p.Glu273Lys	**0.26**		**Likely Oncogenic**
		**INPP4B**	c.2039A>T	p.Glu680Val	**10.8**		**VUS**
		**CDKN2A**	c.65G>C	p.Arg22Pro	**22.1**		**VUS**
		**ATR**	c.3422C>G	p.Ser1141Cys	**0.81**		**VUS**
5	Lung squamous cell carcinoma	**CDKN2A**	c.238C>T	p.Arg80Ter	**5.1**	**2.83**	**Oncogenic**
		**EP300**	c.1586C>T	p.Ser529Phe	**0.56**		**VUS**
6	Lung adenocarcinoma	**ALK**	c.2460_2462delAGG	p.Gly823del	**0.50**	**0.50**	**VUS**
7	Lung adenocarcinoma	**KRAS**	c.35G>A	p.Glu12Asp	**8.8**	**9.02**	**Tier 1**
		**STK11**	c.667G>T	p.Glu223Ter	**9.8**		**Oncogenic**
		**SMARCA4**	c.2342T>C	p.Met781Thr	**10**		**Likely Oncogenic**
		**KEAP1**	c..758T>G	p.Val253Gly	**9.4**		**VUS**
		**MET**	c.3937T>A	p.Tyr1313Asn	**6.1**		**VUS**
		**SMARCB1**	c.1075G>A	p.Ala359Thr	**10**		**VUS**

Suppl. Table S2 (cont.)

**8**	Lung adenocarcinoma	**KRAS**	c.35G>T	p.Gly12Val	**13.6**	**6.7**	**Tier 1**
		**TP53**	c.856G>A	p.Glu286Lys	**6.3**		**Oncogenic**
		**SMARCA4**	c.3728_3729delinsCT	p.Arg1243Pro	**6.4**		**Oncogenic**
		**EP300**	c.3807-4_3837del	splice acceptot	**0.64**		**VUS**
**9**	Lung adenocarcinoma	**EGFR**	c.2235_2249delGGAATTAGAGAAGC	p.Glu746_Ala750del	**29**	**30.8**	**Tier 1**
**(Baseline)**		**TP53**	c.715A>G	p.Asn239Asp	**32.6**		**Oncogenic**
**10**	Lung adenocarcinoma				**NONE**	**0**	
**(Baseline)**							
**11**	Lung adenocarcinoma	**STK11**	c.580G>T	p.Asp194Tyr	**4.6**	**3.73**	**Oncogenic**
		**KEAP1**	c.1379G>T	p.Arg460Met	**4.7**		**VUS**
		**SMO**	c.2081delC	p.Pro694Leufs*82	**1.9**		**VUS**
**12**	Lung adenocarcinoma				**None**	**0**	
**13**	Lung squamous cell carcinoma	**TP53**	c.469G>T	p.V157F	**1.8**	**1.44**	**Oncogenic**
		**PTEN**	c.303_304delCA	p.Lys102Thrfs*4	**1.7**		**Likely Oncogenic**
		**ERBB4**	c.2527delG	p.Asp843Ilefs*8	**0.82**		**VUS**
**14**	Melanoma	**BRAF**	c.1799T>A	p.Val600Glu	**18.7**	**12.9**	**Tier 1**
		**TERT**	c.-146C>T		**7.1**		**Tier 2**
**15**	Melanoma	**BRAF**	c.1798_1799delinsAA	p.Val600Lys	**1.6**	**0.95**	**Tier 1**
		**PTEN**	c.492+1_492+2delGT	Splicing	**1.3**		**Likely Oncogenic**
		**TERT**	c.-124C>T	Promoter	**0.3**		**Likely Oncogenic**
		**FGF9**	c.535A>G	p.Lys179Glu	**0.96**		**VUS**
		**FLT1**	c.3040T>C	p.Ser1014Pro	**0.95**		**VUS**
		**NRG1**	c.1702_1707 delinsAA	p.Asp568Asn	**0.67**		**VUS**
		**RAD54L**	c.110C>T	p.Ser37Phe	**0.85**		**VUS**
**16**	Melanoma	**BRAF**	c.1799T>A	p.Val600Glu	**9.9**	**5.13**	**Tier 1**
		**PTEN**	c.442_443delinsAA	p. Arg148Lys	**0.36**		**Likely Oncogenic**
**17**	Melanoma	**BRAF**	c.1798_1799delinsAA	p.Val600Lys	**2.3**	**2.1**	**Tier 1**
		**TERT**	c.-146C>T	promoter	**0.3**		**Tier 2**
		**TP53**	c.434T>G	p.Leu145Arg	**6.4**		**Oncogenic**
		**MAP2K2**	c.767C>T	p.Ser256Phe	**0.67**		**VUS**
		**JAK3**	c.1838G>A	p.Arg613Gln	**0.73**		**VUS**

Suppl. Table S2 (cont.)

**18**	Melanoma	**TERT**	c.-146C>T	Upstream gene variant	**0.41**	**0.95**	**Tier 2**
		**TP53**	c.833C>T	p.Pro278Leu	**1.4**		**Oncogenic**
		**NF1**	c.3208C>T	p.Gln1070Ter	**0.64**		**Oncogenic**
		**RB1**	c.1848delA	p.Gly617Valfs*6	**1.6**		**Oncogenic**
		**NF1**	c.3656G>A	p.Gly1219Glu	**0.81**		**Likely Oncogenic**
		**ROS1**	c.709C>T	p.Gln237Ter	**0.95**		**Likely Oncogenic**
		**ARID1A**	c.675del	p.Ala226Profs*6	**1.5**		**Likely Oncogenic**
		**APC**	c.5503_5504delinsTA	p.Arg1835Ter	**0.97**		**VUS**
		**ATM**	c.9164G>A	p.Trp3055Ter	**0.68**		**VUS**
		**ALK**	c.3626G>A	p.Arg1209Gln	**2.3**		**VUS**
		**ALK**	c.2194G>A	p.Asp732Asn	**1.6**		**VUS**
		**FGFR3**	c.1552G>A	p.Asp518Asn	**1.3**		**VUS**
		**AR**	c.2685G>A	p.Met895Ile	**1.9**		**VUS**
		**EGFR**	c.347C>T	p.Ser116Phe	**0.86**		**VUS**
		**ROS1**	c.2423C>T	p.Ser808Leu	**0.87**		**VUS**
		**ROS1**	c.3439A>G	p.Thr1147Ala	**1.4**		**VUS**
		**JAK3**	c.2312G>C	p.Arg771Pro	**0.71**		**VUS**
		**JAK3**	c.3359_3360delinsTT		**0.52**		**VUS**
		**CDK12**	c.4226C>T	p.Pro1409Leu	**0.34**		**VUS**
		**CDK12**	c.3041C>T	p.Thr1014Ile	**0.37**		**VUS**
		**FGF9**	c.380C>T	p.Ser127Leu	**0.28**		**VUS**
		**PNPN11**	c.64A>C	p.Thr22Pro	**0.57**		**VUS**
		**FGF23**	c.476C>T	p.Ser159Phe	**0.35**		**VUS**
		**KEAP1**	c.946G>A	p.Val316Met	**0.76**		**VUS**
		**NOTCH1**	c.7598C>T	p.Ser2533Phe	**0.49**		**VUS**
		**SMO**	c.922T>C	p.Ser308Pro	**0.49**		**VUS**
		**ErBB4**	c.2606A>C	p.Glu869Ala	**1.5**		**VUS**
**19**	Melanoma	**TERT**	c.-146C>T	upstream gene variant	**1.1**	**3.40**	**Tier 2**
		**BRAF**	c.1780G>A	p.Asp594Asn	**3.4**		**Oncogenic**
		**GNAS**	c.602G>A	p.Arg201His	**0.37**		**Oncogenic**
		**ERBB4**	c.2608G>A	p.Gly870Arg	**3.7**		**Likely Oncogenic**
		**ERBB4**	c.341G>A	p.Arg114Gln	**4.3**		**Likely Oncogenic**
		**NRAS**	c.64C>A	p.Gln22Lys	**2.9**		**VUS**
		**MSH6**	c.2689A>T	p.Asn897Tyr	**3.5**		**VUS**
		**BTK**	c.1462G>A	p.Glu488Lys	**4**		**VUS**
		**MET**	c.3452G>A	p.Gly1151Glu	**3.1**		**VUS**
		**MET**	c.799G>A	p.Glu267Lys	**2.5**		**VUS**
		**FGF4**	c.581C>T	p.Ser194Leu	**3.7**		**VUS**
		**ERG**	c.1315C>T	p.Pro439Ser	**7.5**		**VUS**
		**KDR**	c.2817+1G>A		**4.1**		**VUS**
**20**	Esophageal adenocarcinoma	**TP53**	c.641 A>G	p.His214Arg	**0.24**	**0.24**	**Oncogenic**
**21**	Colon adenocarcinoma	**KRAS**	c.437C>T	p.Ala146Val	**39.1**	**54.7**	**Tier 1**
**(Baseline)**		**BRAF**	c.1447A>G	p.Lys483Glu	**68.8**		**Tier 2**
		**APC**	c.4326delT	p.Pro1443Leufs*30	**76.3**		**Oncogenic**
		**TP53**	c.524G>A	p.Arg175His	**72.9**		**Oncogenic**
		**SMAD4**	c.667+5G>A	Splicing	**68.6**		**VUS**
		**NOTCH2**	c.952C>T	p.Arg318Cys	**2.5**		**VUS**

Suppl. Table S2 (cont.)

22	Colon adenocarcinoma				None	0	
23	Rectal adenocarcinoma	TP53	c.772G>T	p.Glu258Ter	50	13.7	Oncogenic
		APC	c.1206delT	p.Glu403Lysfs*51	30.5		Oncogenic
		PIK3R1	c.1227delG	p.Asn410Metfs*4	3.1		Likely Oncogenic
		PIK3R1	c.1455delA	p.Ala486Hisfs*6	0.86		Likely Oncogenic
		BCL6	c.1753C>T	p.Arg585Trp	1.1		Likely Oncogenic
		TFRC	c.1190T>G	p.Val397Gly	0.21		VUS
		RB1	c.949C>T	p.Leu317Phe	0.22		VUS
		SMAD4	c.305C>T	p.Pro102Leu	36.5		VUS
		NOTCH1	c.7244_7246delCAC	p.Pro2415del	0.78		VUS
24 (Baseline)	Colon adenocarcinoma				None	0	
25 (Baseline)	Breast cancer	PIK3CA	c.1624G>A	p.GluE542Lys	83.1	35.4	Oncogenic
		TP53	c.673-1G>T	Splicing	36		Oncogenic
		SMARCB1	c.91G>C	p.Glu31Gln	0.5		Likely Oncogenic
		MYC	c.138C>G	p.Phe46Leu	22		VUS
26	Breast cancer	AKT1	c.49G>A	p.Glu17Lys	41.1	9.7	Oncogenic
		CDH1	c.61_86delinsGCT	p.Leu21Alafs*5	28.4		Likely Oncogenic
		FBXW7	c.745A>T	p.Lys249Ter	0.92		Likely Oncogenic
		ROS1	c.5209G>T	p.Glu1737Ter	1.5		Likely Oncogenic
		BAP1	c.421_437+12delinsATGTG	p.Asn142Alafs*8	3.94		Likely Oncogenic
		NOTCH1	c.7244_7246delCAC	p.Pro2415del	0.65		VUS
		ErBB4	c.2912C>G	p.Ala971Gly	0.5		VUS
		GNAS	c.1343A>C	p.Asp448Ala	0.2		VUS
27	Breast cancer	ESR1	c.1606C>G	p.Leu536Val	63.3	27.4	VUS
		FANCI	c.2302T>G	p.Phe768Val	33		VUS
		PIK3CG	c.1630C>T	p.Arg544Ter	1.9		VUS
		CCND2	c.455C>A	p.Ala152Glu	0.9		VUS
		MYCL	c.980T>C	p.Leu327Ser	65.3		VUS
		SLX4	c.608C>G	p.Pro203Arg	0.23		VUS
28 (Post 2)	Breast cancer	PIK3CA	c.3140A>G	p.His1047Arg	21.1	9.5	Oncogenic
		PTEN	c.389G>A	p.Arg130Gln	3.3		Oncogenic
		ESR1	c.1610A>G	p.Tyr537Cys	9.1		Oncogenic
		ESR1	c.1609T>G	p.Tyr537Asp	0.6		VUS
		PIK3CA	c.206T>A	p.Ile69Asn	21.5		VUS
		MTOR	c.6302G>T	p.Trp2101Leu	1.1		VUS
29 (Baseline)	Breast cancer	TP53	c.637C>T	p.Arg213Ter	1.38	1.38	Oncogenic
30 (Baseline)	Breast cancer	TP53	c.536A>T	p.His179Leu	3.9	3.9	Oncogenic
31 (Baseline)	Breast cancer (IBC)	NF1	c.1942G>T	p.Glu648Ter	0.52	1.1	Oncogenic
		CDH1	c.2372_2373delTC	p.Leu791Hisfs*15	1.71		Likely Oncogenic
32 (Baseline)	Breast cancer	PIK3CA	c.1624G>A	p.Glu542Lys	14	9	Oncogenic
		TP53	c.996_999delCCGT	p.Ile332Metfs*12	12.8		Likely Oncogenic
		MRE11	c.1638G>A	p.Met546Ile	0.22		VUS
33 (Post 2)	Breast cancer	TP53	c.396G>C	p.Lys132Asn	1.82	1.24	Oncogenic
		CREBBP	c.4186_4187delCT	p.Leu1396Valfs*4	1.19		Likely Oncogenic
		CCND1	c.860C>T	p.Pro287Leu	0.70		Likely Oncogenic

Supplementary Table S2. Somatic variants in 33 patients with advanced cancer. The somatic variants with VAF % are shown. The mean VAF was calculated for each patient (average of the VAF% detected). IBC, inflammatory breast cancer; VUS, variant of uncertain significance.

Patient ID	Type of cancer	ctFraction OTTER	ctFraction ichorCNA	ctFRACTION Fragle off-target	ctFRACTION Fragle LP-WGS	Mean VAF %
1	Lung adenocarcinoma	11.00	19.48	3.50	1.56	12.33
2	Lung adenocarcinoma	6.00	4.55	5.30	3.69	7.94
3	Lung squamous cell carcinoma	3.00	3.50	9.00	5.19	1.97
4	Lung adenocarcinoma	15.00	25.90	20.80	13.14	15.65
5	Lung squamous cell carcinoma	5.00	0.00	0.62	1.70	2.83
6	Lung adenocarcinoma	0.00	0.00	5.80	3.32	0.5
7	Lung adenocarcinoma	7.00	11.54	19.40	12.67	9.02
8	Lung adenocarcinoma	5.00	7.50	13.20	13.14	6.7
9 (Baseline)	Lung adenocarcinoma	11.00	23.00	14.70	6.60	30.8
10 (Baseline)	Lung adenocarcinoma	6.00	0.00	0.93	0.75	0
11	Lung adenocarcinoma	6.00	5.66	5.50	4.50	3.7
12	Lung adenocarcinoma	5.00	0.00	1.10	2.15	0
13	Lung squamous cell carcinoma	7.00	3.01	6.00	0.45	1.44
14	Melanoma	12.00	12.46	10.11	9.03	12.9
15	Melanoma	2.00	1.90	4.20	3.62	0.95
16	Melanoma	10.00	6.67	9.50	6.74	5.13
17	Melanoma	6.00	14.22	16.50	3.26	2.1
18	Melanoma	0.99	0.00	5.90	4.33	0.95
19	Melanoma	4.30	7.12	4.60	2.02	3.4
20	Esophageal adenocarcinoma	0.00	3.96	2.40	1.61	0.24
21 (Baseline)	Colon adenocarcinoma	49.00	50.84	56.00	52.51	54.7
22	Colon adenocarcinoma	0.00	0.00	15.60	5.22	0
23	Rectal cancer	22.80	47.86	39.80	34.29	13.7
24 (Post 2)	Colon adenocarcinoma	0.00	2.50	4.70	3.01	0
25 (Baseline)	Breast cancer	15.00	30.54	48.70	47.08	35.4
26	Breast cancer	15.00	27.76	43.00	40.03	9.7
27	Breast cancer	26.00	47.42	24.00	26.52	27.4
28 (Post 2)	Breast cancer	18.70	29.30	21.20	18.57	9.5
29 (Baseline)	Breast cancer	2.30	4.10	0.83	1.51	1.38
30 (Baseline)	Breast cancer	4.20	8.50	0.24	0.84	3.9
31 (Baseline)	Breast cancer	0.00	4.10	0.79	1.15	1.1
32 (Baseline)	Breast cancer	13.70	19.77	31.66	26.97	9
33 (Post 2)	Breast cancer	1.65	3.2	4.90	1.77	1.24

Supplementary Table S3. ctFraction estimates by OTTER, ichorCNA, Fragle off-target and Fragle LP-WGS. The ctFraction for the 33 samples (cohort A in Figure 1) are shown. Also, the mean VAF for each sample is indicated.

Suppl. Table S4

Pt ID	Type of tumor	Gene	DNA Variant	AA change	At Baseline VAF (%)	Post 2 VAF (%)	Δ VAF % (Post 2-Baseline)	Mean Δ VAF (%)	Type of variant	Oncogenicity Score
9	Lung adenocarcinoma	TP53	c.715A>G	p.Asn239Asp	32.6	11.8	-20.8	-10.02	Oncogenic	10
		EGFR	c.2235_2249delGGAATTAAGAGAAGC	p.Glu746_Ala750del	29	8.9	-20.1		Tier 1	Tier1
		ERBB4	c.2512C>T	p.Arg838Ter	0	0.23	0.23		Likely oncogenic	3
		AKT1	c.1435A>C	p.Thr479Pro	0	0.6	0.6		VUS	0
10	Lung adenocarcinoma				0	0	0	0.00		
34	Lung adenocarcinoma	SMAD4	c.1486C>T	p.Arg496Cys	0.22	0	-0.22	0.07	Oncogenic	7
		FGFR2	c.910G>A	p.Asp304Asn	0.32	0.27	-0.05		Tier 1	Tier 1
		NOTCH2	c.6892C>T	p.Arg2298Trp	0.43	0.39	-0.04		VUS	1
		NRG1	c.1364C>T	p.Ser455Leu	0	0.22	0.22		VUS	1
		AR	c.1823G>A	p.Arg608Gln	0	0.43	0.43		Oncogenic	7
35	Lung adenocarcinoma				0	0				
36	Lung adenocarcinoma				0	0	0	0.00		
37	Lung adenocarcinoma	TP53	c.734G>T	p.Gly245Val	0.37	0.3	-0.07	-0.07	Oncogenic	9
25	Breast cancer	PIK3CA	c.1624G>A	p.Glu542Lys	83.1	1.29	-81.81	-35.08	Oncogenic	9
		TP53	c.673-1G>T	Splice acceptor variant	36	0	-36		Oncogenic	7
		MYC	c.138C>G	p.Phe46Leu	22	0	-22		VUS	1
		SMARCB1	c.91G>C	p.Glu31Gln	0.50	0	-0.5		Likely Oncogenic	3
28	Breast cancer	PIK3CA	c.3140A>G	p.His1047Arg	24.8	21.1	-3.7	-1.67	Oncogenic	9
		PTEN	c.389G>A	p.Arg130Gln	6.6	3.3	-3.3		Oncogenic	9
		ESR1	c.1610A>G	p.Tyr537Cys	10.5	9.1	-1.4		Oncogenic	7
		ESR1	c.1609T>G	p.Tyr537Asp	0.87	0.6	-0.27		VUS	2
		PIK3CA	c.206T>A	p.Ile69Asn	24.8	21.5	-3.3		VUS	2
		MTOR	c.6302G>T	p.Trp2101Leu	0.58	1.1	0.52		VUS	2
		CTNNB1	c.2338G>A	p.Asp780Asn	0.21	0	-0.21		VUS	1
29	Breast cancer	TP53	**c.637C>T**	**p.Arg213Ter**	1.38	0	-1.38	-1.38	Oncogenic	9
30	Breast cancer	TP53	c.536A>T	p.His179Leu	3.9	0	-3.9	-3.90	Oncogenic	9

Suppl. Table S4 (cont.)

31	Breast cancer	NF1	c.1942G>T	p.Glu648Ter	0.52	0	-0.52	-1.11	Oncogenic	7
		CDH1	c.2372_2373delTC	p.Leu791Hisfs*15	1.7	0	-1.7		Likely oncogenic	3
32	Breast cancer	PIK3CA	c.1624G>A	p.GluE542Lys	14	0	-14	-5.31	Oncogenic	9
		TP53	c.996_999delCCGT	p.Ile332Metfs*12	12.8	0	-12.8		Likely oncogenic	3
		MRE11	c.1638G>A	p.Met546Ile	0.22	0	-0.22		VUS	0
		FGFR2	c.910G>A	p.Asp304Asn	0	0.2	0.2		Likely Oncogenic	3
		PPP2R2A	c.1183C>T	p.Arg395Cys	0	0.27	0.27		VUS	1
33	Breast cancer	TP53	c.396G>C	p.Lys132Asn	2.5	1.82	-0.68	-0.60	Oncogenic	7
		CREBBP	c.4186_4187delCT	p.Leu1396Valfs*4	2.3	1.19	-1.11		Likely oncogenic	3
		CCND1	c.860C>T	p.Pro287Leu	1.4	0.70	-0.7		Likely Oncogenic	4
		FGFR2	c.910G>A	p.Asp304Asn	0.23	0	-0.23		Likely oncogenic	3
		CDK6	c.151G>A	p.Glu51Lys	0.26	0	-0.26		VUS	2
38	Breast cancer	TP53	c.853G>A	p.Glu285Lys	5.20	0.54	-4.66	-4.66	Oncogenic	8
21	Colon adenocarcinoma	APC	c.4326delT	p.Pro1443Leufs*30	76.3	1.33	-74.97	-53.74	Oncogenic	6
		TP53	c.524G>A	p.Arg175His	72.9	0.71	-72.19		Oncogenic	8
		SMAD4	c.667+5G>A	Splicing	68.6	0.74	-67.86		VUS	2
		NOTCH2	c.952C>T	p.Arg318Cys	2.5	0	-2.5		VUS	1
		BRAF	c.1447A>G	p.Lys483Glu	68.8	2.11	-66.69		Tier 2	Tier 2
		KRAS	c.437C>T	p.Ala146Val	39.1	0.88	-38.22		Tier 1	Tier 1
24	Colon adenocarcinoma				0	0	0	0.00		
39	Rectal adenocarcinoma	TP53	c.380C>A	p.Ser127Tyr	0.40	0.5	0.1	0.16	Oncogenic	8
		TP53	c.848G>A	p.Arg283His	0	0.21	0.21		Oncogenic	6
40	Rectal adenocarcinoma	APC	c.3340C>T	p.Arg1114Ter	0.34	0.12	-0.22	-0.21	Oncogenic	8
		KRAS	c.437C>T	p.Ala146Val	0.31	0.11	-0.2		Tier 1	Tier 1
		CHEK2	c.1239_1240delinsCdelG	p.Leu413Phefs*24	0.49	0.27	-0.22		Likely Oncogenic	3
41	Colon adenocarcinoma	TP53	c.818G>A	p.Arg273His	10.3	1.4	-8.9	-7.42	Oncogenic	10
		KRAS	c.34G>A	p.Gly12Ser	9.82	1.8	-8.02		Tier 1	Tier 1
		PIK3CA	c.1624G>A	p.Glu542Lys	0.24	0	-0.24		Oncogenic	9
		APC	c.3956delC	p.Pro1319Leufs*2	15.3	2.8	-12.5		Oncogenic	8
		MTOR	c.7453G>T	p.Asp2485Tyr	0.37	0	-0.37		VUS	1
42	Colon adenocarcinoma				0	0	0	0.00		

Supplementary Table S4. Somatic variants were detected at baseline and after 2 cycles of treatment in a cohort of 20 patients (cohort B in Figure 1) with advanced solid tumors. The VAF (%) are shown. The ΔVAF (%) for each variant and the Mean ΔVAF for each patient are indicated. VUS, variant of uncertain significance.

Pt ID	Type of tumor	ichorCNA (%) Baseline	ichorCNA (%) Post 2	Fragle off- target (%) Baseline	Fragle off- target (%) Post 2	ichorCNA (%) Δ ctFraction ichor	Fragle off-target (%) Δ ctFraction Fragle	Mean Δ VAF (%)
9	Lung adenoc.	23.0	12.8	14.70	4.70	-10.17	-10	-10.02
10	Lung adenoc.	0.0	0.0	0.93	0.50	0	-0.43	0
34	Lung adenoc.	0.0	0.0	1.37	5.10	0	3.73	0.07
35	Lung adenoc.	0.0	0.0	3.95	2.98	0	-0.97	0
36	Lung adenoc.	0.0	0.0	0.72	1.09	0	0.37	0
37	Lung adenoc.	0.0	0.0	1.66	2.50	0	0.84	-0.07
25	Breast cancer	30.54	0.0	48.70	1.44	-30.54	-47.26	-35.08
28	Breast cancer	33.7	29.3	20.20	21.20	-4.44	1	-1.67
29	Breast cancer	4.1	4.8	0.83	1.00	0.7	0.17	-1.38
30	Breast cancer	8.5	0.0	0.24	0.90	-8.53	0.66	-3.9
31	Breast cancer (IBC)	4.1	0.0	0.79	0.14	-4.1	-0.65	-1.11
32	Breast cancer	19.77	0.0	31.66	1.10	-19.77	-30.56	-5.31
33	Breast cancer	6.8	3.2	4.70	4.90	-3.6	0.2	-0.6
38	Breast cancer	6.8	0.0	8.40	1.20	-6.8	-7.2	-4.66
21	Colon adenoc.	50.84	2.6	56.00	6.50	-48.24	-49.5	-53.74
24	Colon adenoc.	0.0	2.5	1.00	4.70	2.5	3.7	0
39	Rectal adenoc.	1.9	0.0	1.30	2.00	-1.9	0.7	0.16
40	Rectal adenoc.	0.0	0.0	2.50	2.20	0	-0.3	-0.21
41	Colon adenoc.	9.5	3.9	28.80	14.80	-5.6	-14	-7.42
42	Colon adenoc.	0.0	0.0	5.10	5.10	0	0	0

Supplementary Table S5. ctFraction estimates by ichorCNA and Fragle off-target at baseline and after 2 cycles of treatment (Post 2) in cohort B (n=20 patients). The differences in ctFraction (ΔctFraction) using ichorCNA and Fragle off-target and the average ΔVAF are indicated.

FCCC ctDNA Assay				
AKT1	CHEK1	FGF10	MDM4	PIK3R1
AKT2	CHEK2	FGF14	MET	PMS2
AKT3	CREBBP	FGF23	MLH1	PPP2R2A
ALK	CSF1R	FGFR1	MLLT3	PTCH1
APC	CTNNB1	FGFR2	MPL	PTEN
AR	DDR2	FGFR3	MRE11A	PTPN11
ARID1A	DNMT3A	FGFR4	MSH2	RAD51
ATM	EGFR	FLT1	MSH3	RAD51B
ATR	EP300	FLT3	MSH6	RAD51C
BAP1	ERBB2	FOXL2	MTOR	RAD51D
BARD1	ERBB3	GEN1	MUTYH	RAD54L
BCL2	ERBB4	GNA11	MYC	RB1
BCL6	ERCC1	GNAQ	MYCL1	RET
BRAF	ERCC2	GNAS	MYCN	RICTOR
BRCA1	ERG	HNF1A	MYD88	ROS1
BRCA2	ESR1	HRAS	NBN	RPS6KB1
BRIP1	EZH2	IDH1	NF1	SLX4
BTK	FAM175A	IDH2	NOTCH1	SMAD4
CARD11	FANCI	INPP4B	NOTCH2	SMARCA4
CCND1	FANCL	JAK2	NOTCH3	SMARCB1
CCND2	FBXW7	JAK3	NPM1	SMO
CCNE1	FGF1	KDR	NRAS	SRC
CD79A	FGF2	KEAP1	NRG1	STK11
CD79B	FGF3	KIT	PALB2	TERT
CDH1	FGF4	KMT2A (MLL)	PDGFRA	TET2
CDK12	FGF5	KRAS	PDGFRB	TP53
CDK4	FGF6	MAP2K1	PIK3CA	TSC1
CDK6	FGF7	MAP2K2	PIK3CB	TSC2
CDKN2A	FGF8	MCL1	PIK3CD	VHL
CEBPA	FGF9	MDM2	PIK3CG	XRCC2

Supplementary Table S6. Gene panel in FCCC ctDNA assay. The assay includes 5389 single-stranded DNA probes targeted at 150 genes. This assay can detect SNV and indels in the genes indicated in the table.
